# Supplementary material for: Treatment with a Cholecystokinin Receptor Antagonist, Proglumide, Improves Efficacy of Immune Checkpoint Antibodies in Hepatocellular Carcinoma
Source: Int J Mol Sci. 2023 Feb 11;24(4):3625. doi: 10.3390/ijms24043625 (PMC9965856; doi:10.3390/ijms24043625)
Supplement: Supplementary file 1 [file ijms-24-03625-s001.zip › ijms-2078580-supplementary.pdf]

Supplemental Materials Table of Contents

Supplemental Figure S1. Dissociation curves analysis of qPCR amplification for mouse CCK-BR and gastrin genes.

Supplemental Figure S2. Representative dissociation curve analysis of qPCR amplification for different genes using total RNA from mouse tumors.

Supplemental Figure S3. Gene interaction network map for hepatic fibrosis signaling pathway.

Supplemental Table S1: Raw Ct Data and calculation of relative expression data from the raw Ct data.

Supplemental Table S2: Effect of Proglumide treatment on gene expression in Hepa1-6 cells after 8 hrs and 24 hrs at 20 nM and 40 nM dose.

Supplemental Table S3: Up-regulated genes by RNAseq control versus proglumide treated HepG2 human HCC cells.

Supplemental Table S4: Down-regulated genes by RNAseq control versus proglumide treated HepG2 human HCC cells.

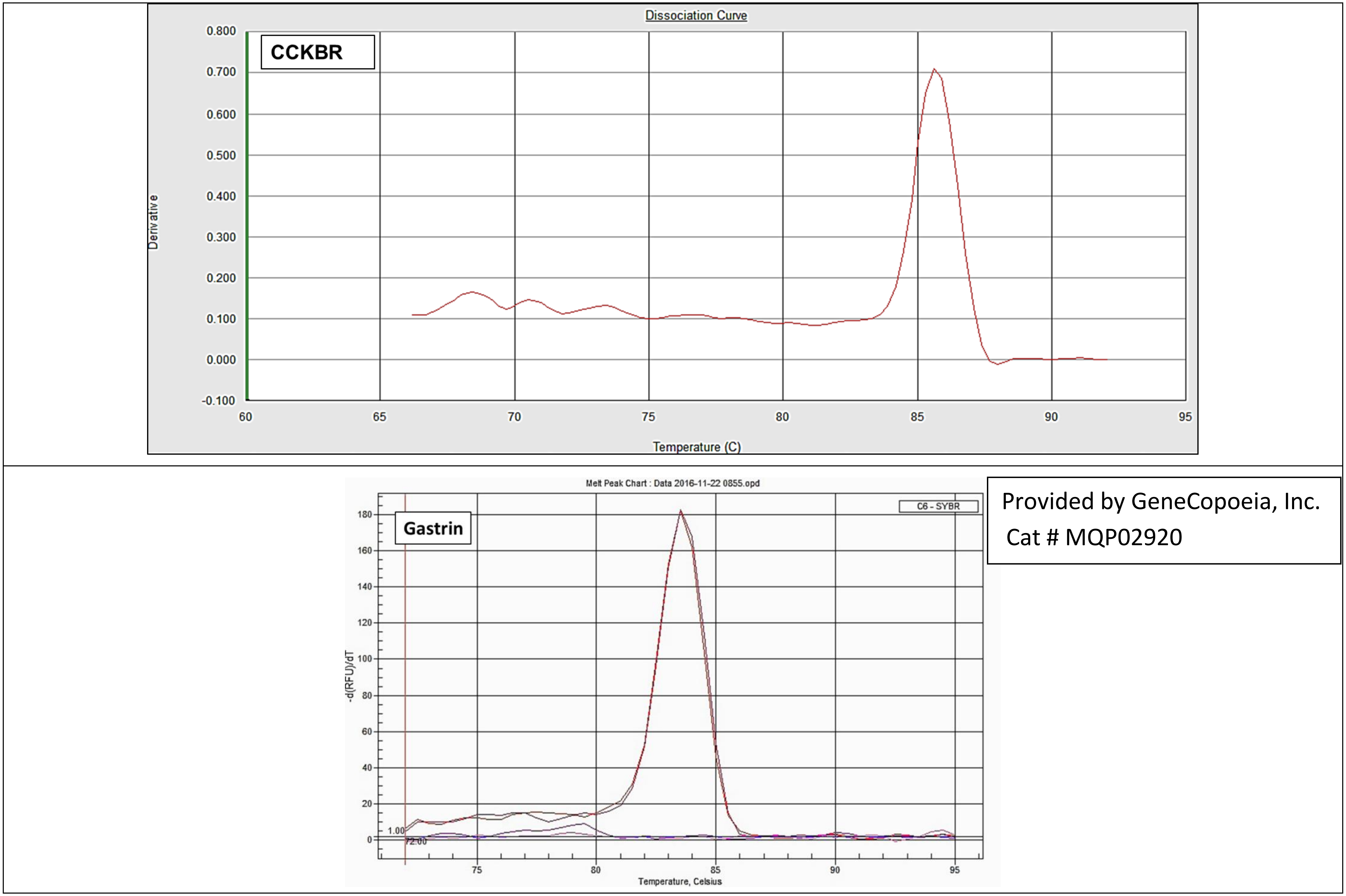

Supplemental Figure S1. Dissociation curve analysis of qPCR amplification for mouse CCK-BR and mouse gastrin (for Figure 1).

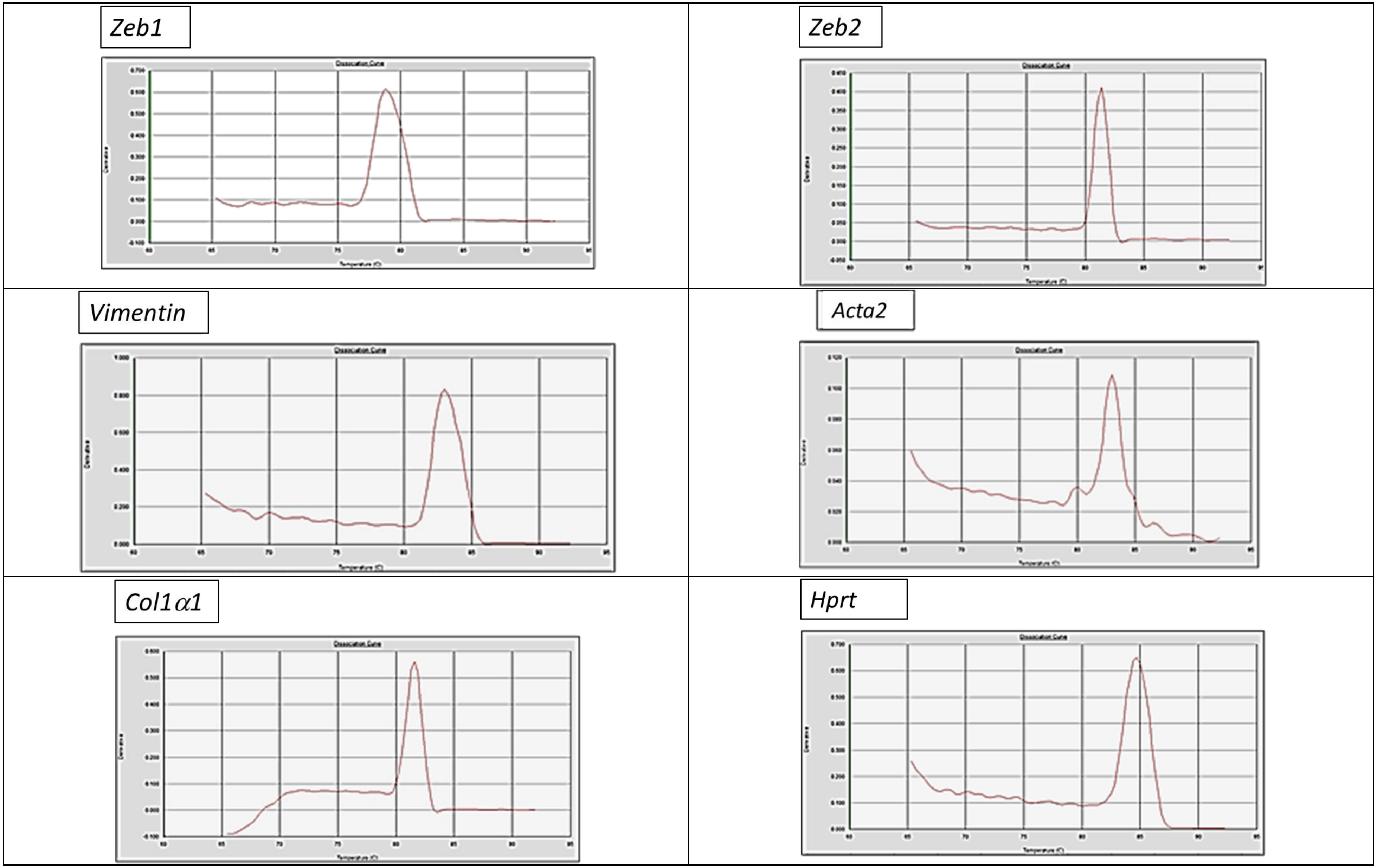

Supplemental Figure S2. Representative dissociation curve analysis of qPCR amplification for different genes using total RNA from mouse tumors (Figure 5). The figure shows single peak in the dissociation curve analysis in each case indicative of single amplicon formed following PCR amplification(specificity) in case of all genes.

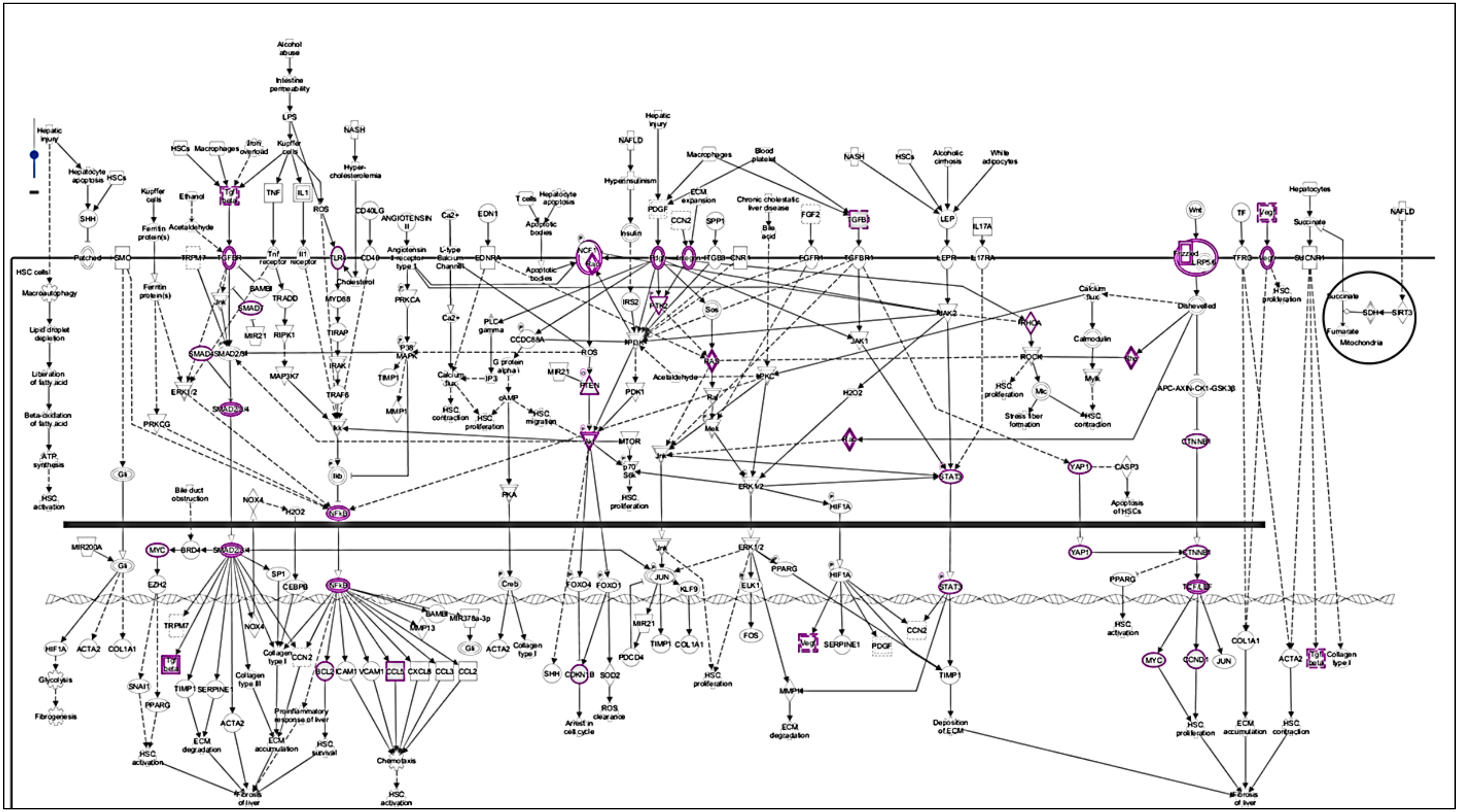

Supplemental Figure S3. Gene interaction network map for hepatic fibrosis signaling pathway. This network consist of the following genes: (*Akt1, Bcl2, Ccl5, Ccnd1, Cdkn1b, Ctnnb1, Flt1, Fzd7, Hras, Itgb1, Kdr, Lef1, Myc, Nfkb1, Nras, Pdgfra, Pten, Ptk2, Rac1, Rhoa, Smad4, Smad7, Stat3, Tcf4, Tgfb1, Tgfbr2, Tlr4, Vegfa, Yap1*) which are part of Qiagen RT2 Profiler PCR Array Mouse Liver Cancer (Cat. #330231) as described in Materials & Methods. The genes are highlighted as pink circles and squares. The figure shows that hepatic stellate cells contribute to fibrogenesis during liver injury transitioning them from quiescent to proliferative, migratory and fibrogenic phenotype. In the presence of proglumide, this fibrosis phenotype is greatly diminished. Gene interaction network map has been generated using IPA (Qiagen).

Suppplemental Table S1: Raw Ct Data and calculation of Relative expression data from the raw Ct data

Ct data

Gene	Control1	Control2	Control3	Control4	Progl1	Progl2	Progl3	Progl4	PD-1Ab-1	PD-1Ab-2	PD-1Ab-3	PD-1Ab-4	COMBO-1	COMBO-2	COMBO-3	COMBO-4
Zeb-1	21.985	22.5252	21.8594	20.8702	20.6484	23.5412	25.3259	22.025	27.806	25.7773	25.0329	24.5361	20.8736	22.2171	22.3209	23.107
Zeb-2	23.6355	23.4742	22.4843	21.9316	21.3468	25.0289	26.3819	23.0256	27.2923	27.6724	25.4134	26.394	20.7067	23.1684	23.7626	22.8486
Vimentin	20.7373	20.9688	19.9141	19.6446	19.1269	22.9789	24.1989	21.1025	26.9766	25.2694	24.3401	24.2616	18.0991	21.3182	23.102	21.303
Acta2	26.0525	24.7087	23.9509	22.0198	23.4871	26.0466	27.426	25.7459	29.1082	30.5372	27.1597	28.0108	23.0235	25.5081	25.1794	25.0056
Hprt	25.1296	25.1959	24.4028	23.214	22.7321	26.4607	27.5783	24.1545	30.0551	30.3617	27.7509	27.6681	21.2653	24.2266	24.3483	24.6616

Fold Change (Relative expression)

Gene	Control1	Control2	Control3	Control4	Progl1	Progl2	Progl3	Progl4	PD-1Ab-1	PD-1Ab-2	PD-1Ab-3	PD-1Ab-4	COMBO-1	COMBO-2	COMBO-3	COMBO-4
Zeb-1	1	0.72	0.66	0.57	0.48	0.8	0.54	0.49	0.54	2.71	0.74	0.99	0.15	0.46	0.46	0.33
Zeb-2	1	1.17	1.34	0.86	0.93	0.96	0.82	0.78	2.41	2.29	1.79	0.86	0.52	0.74	0.53	1.21
Vimentin	1	0.9	1.07	0.57	0.58	0.53	0.5	0.39	0.4	1.63	0.51	0.5	0.43	0.36	0.11	0.49
Acta2	1	2.66	2.59	4.34	1.12	2.53	2.11	0.66	3.65	1.68	2.86	1.5	0.56	0.8	1.07	1.49

Ct data

	Control1	Control2	Control3	Control4	Prog1	Prog2	Prog3	Prog4	PD-1Ab-1	PD-1Ab-2	PD-1Ab-3	PD-1Ab-4	COMBO1	COMBO2	COMBO3	COMBO4
Col1(α)1	28.2784	28.0657	26.3864	26.1612	32.1462	29.4827	30.0847	29.5289	26.032	30.4616	30.6332	28.1022	24.0922	26.906	26.8582	24.4892
Hprt	26.6756	26.9128	24.6408	24.7215	30.6653	27.2431	28.5636	27.318	24.0065	28.7814	28.6616	26.0213	21.6744	24.6568	25.0439	23.3933

Col1(α)1 was analysed at different time on the the same RNA but c-DNA prepared again newly, thus along with Col1(α)1, Hprt was also run again as reference.

Fold Change (Relative expression)

	Control1	Control2	Control3	Control 4	Pog1	Pog 2	Pog 3	Pog 4	PD-1Ab-1	PD-1Ab-2	PD-1Ab-3	PD-1Ab-4	COMBO1	COMBO2	COMBO3	COMBO4
Col1(α)1	1	1.366	0.91	1.11	1.09	0.638	1.058	0.6564	0.746	1.118	1.29	0.718	0.5684	0.6488	0.87	0.71

Effect of Proglumide , PD1-Ab and Combo on RIL-175 (Coumns labeled shown in the order as presented in Fig5)

Gene	Control	PD-1Ab	Prog	COMBO	P value Combo
Zeb-1	0.74 ± 0.10	1.24 ± 0.49	0.59 ± 0.09	0.35 ± 0.07	0.017
Zeb-2	1.09 ± 0.10	1.83 ± 0.35	0.87 ± 0.043	0.75 ± 0.16	NS
Vimentin	0.89 ± 0.11	0.76 ± 0.29	0.5 ± 0.04 (P=0.018)	0.36 ± 0.08	0.01
Col1(α)1	1.10 ± 0.0.099	0.96 ± 0.14	0.86 ± 0.12	0.70 ± 0.0.06	0.01
Acta2	2.64 ± 0.68	2.42 ± 0.50	1.60 ± 0.43	0.98 ± 0.19	0.05
(p value)*					

Supplemental Table S2: Effect of Proglumide treatment on gene expression in Hepa1-6 cells after 8 and 24 hrs at 20 nM and 40 nM dose

Gene	Control (8hr)	20nM (8hr)	40nM (8hr)
Zeb-1	1	1.07	0.96
Zeb-2	1	1.02	0.9
Vimentin	1	0.91	0.95
Col1(α)1	1	0.39	0.44
Acta2	1	0.84	0.75

Gene	Control (24hr)	20nM (24hr)	40nM (24hr)
Zeb-1	1	0.83	0.91
Zeb-2	1	0.98	1.1
Vimentin	1	0.86	0.92
Col1(α)1	1	0.46	0.52
Acta2	1	0.77	0.74

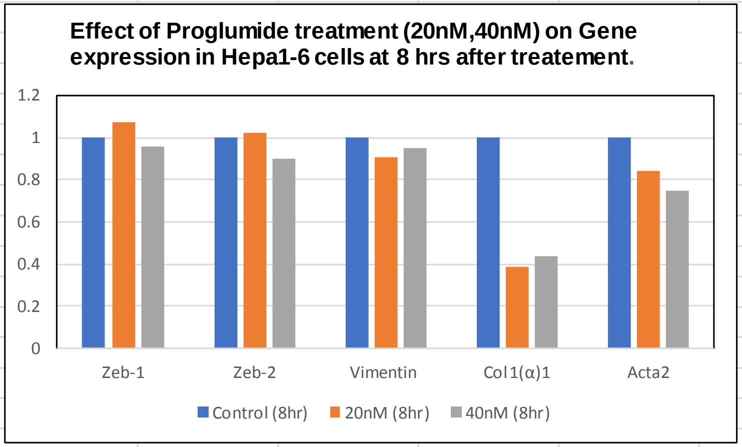

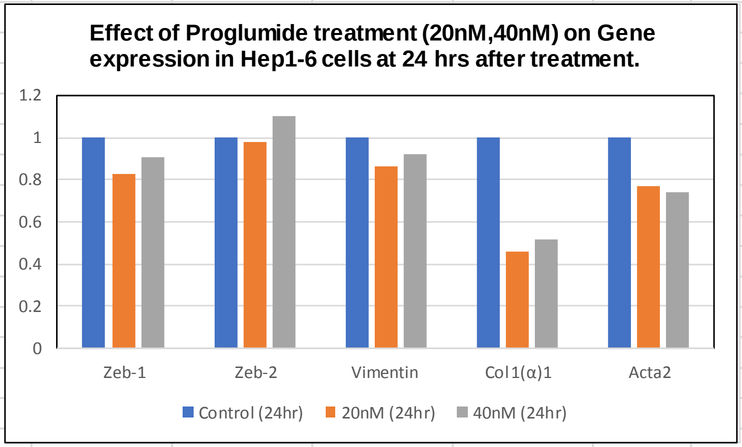

Supplemental Table S3: Up-Regulated Genes Control Versus Proglumide

	A	B	C	D	E	F	G	H	I
1	**gene_id**	**Proglumide**	**Control**	**log2FoldChange**	**pvalue**	**gene_name**	**gene_chr**	**gene_biotype**	**gene_description**
2	ENSG00000236675	382.931409	20.7285356	4.199114493	3.22E-15	MTX1P1	1	unprocessed_pseudo	metaxin 1 pseudogene 1 [Source:HGNC Symbol;Acc:HGNC:7505]
3	ENSG00000275895	823.605186	88.8401492	3.210840387	7.05E-12	U2AF1L5	21	protein_coding	U2 small nuclear RNA auxiliary factor 1 like 5 [Source:HGNC Symbol;A
4	ENSG00000226124	28.3663029	1.39E-17	7.832514884	1.09E-06	FTCDNL1	2	protein_coding	formiminotransferase cyclodeaminase N-terminal like [Source:HGNC
5	ENSG00000166415	37.4836908	4.93452536	2.893639573	0.0001213	WDR72	15	protein_coding	WD repeat domain 72 [Source:HGNC Symbol;Acc:HGNC:26790]
6	ENSG00000182021	20.2619587	0.98600522	4.196191369	0.00024803	AL591379.1	9	transcribed_unproces	methylenetetrahydrofolate dehydrogenase (NADP+ dependent) 1 like
7	ENSG00000212901	222.870589	70.0847782	1.667242178	0.00031972	KRTAP3-1	17	protein_coding	keratin associated protein 3-1 [Source:HGNC Symbol;Acc:HGNC:1677
8	ENSG00000279267	14.1837014	1.39E-17	6.838830296	0.00051726	AL078621.3	2	TEC	TEC
9	ENSG00000224739	18.2358728	0.98600522	4.045170767	0.00057541	AC016735.1	2	lincRNA	novel transcript
10	ENSG00000178429	28.3663029	3.94739696	2.806128887	0.00063679	RPS3AP5	10	processed_pseudoge	ribosomal protein S3A pseudogene 5 [Source:HGNC Symbol;Acc:HGNC
11	ENSG00000196620	13.1706587	1.39E-17	6.732884245	0.00085757	UGT2B15	4	protein_coding	UDP glucuronosyltransferase family 2 member B15 [Source:HGNC Syn
12	ENSG00000120278	29.379346	4.93452536	2.543489306	0.00118483	PLEKHG1	6	protein_coding	pleckstrin homology and RhoGEF domain containing G1 [Source:HGNC
13	ENSG00000231023	50.6532515	12.8315343	1.970379552	0.00123951	LINC00326	6	lincRNA	long intergenic non-protein coding RNA 326 [Source:HGNC Symbol;Ac
14	ENSG00000272129	16.209787	0.98600522	3.87647294	0.00136958	AL359715.3	6	lincRNA	novel transcript
15	ENSG00000216819	12.157616	1.39E-17	6.618536883	0.00143701	TUBB2BP1	6	unprocessed_pseudo	tubulin beta 2B class IIb pseudogene 1 [Source:HGNC Symbol;Acc:HG
16	ENSG00000273102	12.157616	1.39E-17	6.618536883	0.00143701	AP000569.1	21	lincRNA	novel transcript
17	ENSG00000266983	19.2489157	1.97313462	3.206094843	0.00159793	AC011444.1	19	antisense	uncharacterized LOC100128568 [Source:NCBI gene;Acc:100128568]
18	ENSG00000285219	25.3271738	3.94739696	2.643389466	0.0016487	AL591485.1	6	bidirectional_promot	uncharacterized LOC100506207 [Source:NCBI gene;Acc:100506207]
19	ENSG00000122674	375.840107	144.119137	1.382071172	0.00171659	CCZ1	7	protein_coding	CCZ1 homolog, vacuolar protein trafficking and biogenesis associate
20	ENSG00000076641	15.1967442	0.98600522	3.784099339	0.00213372	PAG1	8	protein_coding	phosphoprotein membrane anchor with glycosphingolipid microdomai
21	ENSG00000261744	15.1967442	0.98600522	3.784099339	0.00213372	AC116552.1	16	antisense	novel transcript, antisense to ZFPM1
22	ENSG00000240356	146.892351	53.3036565	1.460261063	0.00214681	RPL23AP7	2	transcribed_processe	ribosomal protein L23a pseudogene 7 [Source:HGNC Symbol;Acc:HG
23	ENSG00000173156	35.4576046	7.89590589	2.149095693	0.00220539	RHOD	11	protein_coding	ras homolog family member D [Source:HGNC Symbol;Acc:HGNC:670]
24	ENSG00000251580	62.8097692	18.7542855	1.736945698	0.00223194	LINC02482	4	lincRNA	long intergenic non-protein coding RNA 2482 [Source:HGNC Symbol;
25	ENSG00000188092	135.748877	49.3551573	1.457303189	0.00235452	GPR89B	1	protein_coding	G protein-coupled receptor 89B [Source:HGNC Symbol;Acc:HGNC:138
26	ENSG00000197099	18.2358728	1.97313462	3.128609348	0.00236482	AC068631.1	3	antisense	novel transcript, antisense to KNG1, HRG, FETUB, AHSG and DNAJB1
27	ENSG00000181781	18.2358728	1.97313462	3.128609348	0.00236482	ODF3L2	19	protein_coding	outer dense fiber of sperm tails 3 like 2 [Source:HGNC Symbol;Acc:H
28	ENSG00000173702	49.6402084	13.8186597	1.835384837	0.00241222	MUC13	3	protein_coding	mucin 13, cell surface associated [Source:HGNC Symbol;Acc:HGNC:7
29	ENSG00000260971	11.1445735	1.39E-17	6.494340146	0.00243493	AC119674.1	1	lincRNA	novel transcript
30	ENSG00000220685	11.1445735	1.39E-17	6.494340146	0.00243493	AL139094.1	6	processed_pseudogene	transcription factor B2, mitochondrial (TFB2M) pseudogene
31	ENSG00000006606	11.1445735	1.39E-17	6.494340146	0.00243493	CCL26	7	protein_coding	C-C motif chemokine ligand 26 [Source:HGNC Symbol;Acc:HGNC:106
32	ENSG00000253893	11.1445735	1.39E-17	6.494340146	0.00243493	FAM85B	8	antisense	family with sequence similarity 85 member B [Source:HGNC Symbol;
33	ENSG00000276717	11.1445735	1.39E-17	6.494340146	0.00243493	AL136097.3	9	unprocessed_pseudo	protease, serine, 12 (neurotrypsin, motopsin) (PRSS12) pseudogene
34	ENSG00000123171	11.1445735	1.39E-17	6.494340146	0.00243493	CCDC70	13	protein_coding	coiled-coil domain containing 70 [Source:HGNC Symbol;Acc:HGNC:25
35	ENSG00000231721	89.1488913	29.6126602	1.585877322	0.00255608	LINC-PINT	7	processed_transcript	long intergenic non-protein coding RNA, p53 induced transcript [Sour
36	ENSG00000160182	92.1880208	31.58691	1.541443368	0.0031084	TFF1	21	protein_coding	trefoil factor 1 [Source:HGNC Symbol;Acc:HGNC:11755]
37	ENSG00000177455	50.6532515	14.8057849	1.765785373	0.00314483	CD19	16	protein_coding	CD19 molecule [Source:HGNC Symbol;Acc:HGNC:1633]
38	ENSG00000240476	45.5880358	12.8315343	1.818770987	0.00319623	LINC00973	3	lincRNA	long intergenic non-protein coding RNA 973 [Source:HGNC Symbol;Ac
39	ENSG00000279332	14.1837014	0.98600522	3.685404125	0.00334606	AC090772.4	18	TEC	TEC
40	ENSG00000272906	17.2228299	1.97313462	3.046724811	0.00351516	AL353708.3	1	lincRNA	novel transcript, antisense to TOR1AIP1

	gene_id	Proglumide	Control	log2FoldChange	pvalue	gene_name	gene_chr	gene_biotype	gene_description
41	ENSG00000156127	17.2228299	1.97313462	3.046724811	0.00351516	BATF	14	protein_coding	basic leucine zipper ATF-like transcription factor [Source:HGNC Syn
42	ENSG00000274528	17.2228299	1.97313462	3.046724811	0.00351516	AC090970.2	15	sense_intronic	novel transcript
43	ENSG00000164266	35.4576046	8.88303193	1.98167046	0.00406065	SPINK1	5	protein_coding	serine peptidase inhibitor, Kazal type 1 [Source:HGNC Symbol;Acc:I
44	ENSG00000135625	10.1315311	1.39E-17	6.358435969	0.00417417	EGR4	2	protein_coding	early growth response 4 [Source:HGNC Symbol;Acc:HGNC:3241]
45	ENSG00000158428	10.1315311	1.39E-17	6.358435969	0.00417417	CATIP	2	protein_coding	ciliogenesis associated TTC17 interacting protein [Source:HGNC Sy
46	ENSG00000231357	10.1315311	1.39E-17	6.358435969	0.00417417	AC006028.1	7	processed_pseudogen	prefoldin subunit 4 (PFDN4) pseudogene
47	ENSG00000257660	10.1315311	1.39E-17	6.358435969	0.00417417	AC117498.2	12	lincRNA	uncharacterized LOC100506125 [Source:NCBI gene;Acc:100506125
48	ENSG00000256061	10.1315311	1.39E-17	6.358435969	0.00417417	DNAAF4	15	protein_coding	dynein axonemal assembly factor 4 [Source:HGNC Symbol;Acc:HGN
49	ENSG00000273199	10.1315311	1.39E-17	6.358435969	0.00417417	AP000692.2	21	antisense	novel transcript, antisense to MORC3
50	ENSG00000251661	27.3532599	5.92165277	2.18376506	0.00462106	AC136475.1	11	antisense	novel transcript, antisense to IFITM3
51	ENSG00000236778	19.2489157	2.96026697	2.650053167	0.00484911	INTS6-AS1	13	antisense	INTS6 antisense RNA 1 [Source:HGNC Symbol;Acc:HGNC:42691]
52	ENSG00000237686	123.592359	48.3680324	1.351153843	0.00493237	AL109615.3	6	antisense	uncharacterized LOC101929705 [Source:NCBI gene;Acc:101929705
53	ENSG00000185467	16.209787	1.97313462	2.959911521	0.00524731	KPNA7	7	protein_coding	karyopherin subunit alpha 7 [Source:HGNC Symbol;Acc:HGNC:2183
54	ENSG00000225611	13.1706587	0.98600522	3.579458074	0.00528154	LINC02158	3	antisense	long intergenic non-protein coding RNA 2158 [Source:HGNC Symbo
55	ENSG00000249249	13.1706587	0.98600522	3.579458074	0.00528154	AC010226.1	5	antisense	uncharacterized LOC101927100 [Source:NCBI gene;Acc:10192710
56	ENSG00000187260	24.3141307	4.93452536	2.271743569	0.00528628	WDR86	7	protein_coding	WD repeat domain 86 [Source:HGNC Symbol;Acc:HGNC:28020]
57	ENSG00000212724	24.3141307	4.93452536	2.271743569	0.00528628	KRTAP2-3	17	protein_coding	keratin associated protein 2-3 [Source:HGNC Symbol;Acc:HGNC:18
58	ENSG00000136235	74.9662871	26.6512854	1.487610685	0.00570156	GPNMB	7	protein_coding	glycoprotein nmb [Source:HGNC Symbol;Acc:HGNC:4462]
59	ENSG00000224945	31.4054322	7.89590589	1.974662755	0.00591186	AL353150.1	9	sense_intronic	novel transcript
60	ENSG00000180596	60.7836829	20.7285356	1.546255463	0.00602796	HIST1H2BC	6	protein_coding	histone cluster 1 H2B family member c [Source:HGNC Symbol;Acc:H
61	ENSG00000280099	21.2750017	3.94739696	2.393199599	0.00607288	AL603750.1	1	TEC	TEC
62	ENSG00000205238	46.6010789	14.8057849	1.645799909	0.00637015	SPDYE2	7	protein_coding	speedy/RINGO cell cycle regulator family member E2 [Source:HGNC
63	ENSG00000161031	33.4315184	8.88303193	1.897088672	0.00648527	PGLYRP2	19	protein_coding	peptidoglycan recognition protein 2 [Source:HGNC Symbol;Acc:HGN
64	ENSG00000134240	18.2358728	2.96026697	2.572567673	0.00696616	HMGCS2	1	protein_coding	3-hydroxy-3-methylglutaryl-CoA synthase 2 [Source:HGNC Symbol;A
65	ENSG00000232060	18.2358728	2.96026697	2.572567673	0.00696616	SLC4A1APP1	9	processed_pseudogen	solute carrier family 4 member 1 adaptor protein pseudogene 1 [So
66	ENSG00000163220	9.11848879	1.39E-17	6.208385228	0.00724341	S100A9	1	protein_coding	S100 calcium binding protein A9 [Source:HGNC Symbol;Acc:HGNC:1
67	ENSG00000170075	9.11848879	1.39E-17	6.208385228	0.00724341	GPR37L1	1	protein_coding	G protein-coupled receptor 37 like 1 [Source:HGNC Symbol;Acc:HGN
68	ENSG00000251574	9.11848879	1.39E-17	6.208385228	0.00724341	AC099520.1	5	lincRNA	uncharacterized LOC105379109 [Source:NCBI gene;Acc:105379109
69	ENSG00000223548	9.11848879	1.39E-17	6.208385228	0.00724341	AC034228.1	5	antisense	novel transcript
70	ENSG00000173261	9.11848879	1.39E-17	6.208385228	0.00724341	PLAC8L1	5	protein_coding	PLAC8 like 1 [Source:HGNC Symbol;Acc:HGNC:31746]
71	ENSG00000253582	9.11848879	1.39E-17	6.208385228	0.00724341	AC090579.1	8	antisense	uncharacterized LOC105375700 [Source:NCBI gene;Acc:105375700
72	ENSG00000181074	9.11848879	1.39E-17	6.208385228	0.00724341	OR52N4	11	protein_coding	olfactory receptor family 52 subfamily N member 4 (gene/pseudoge
73	ENSG00000236514	9.11848879	1.39E-17	6.208385228	0.00724341	AL135791.1	10	antisense	novel transcript
74	ENSG00000259013	9.11848879	1.39E-17	6.208385228	0.00724341	AL118558.2	14	processed_pseudogen	NADH dehydrogenase (ubiquinone) 1 alpha subcomplex, 4, 9kDa (N
75	ENSG00000271623	9.11848879	1.39E-17	6.208385228	0.00724341	AC138894.3	16	processed_pseudogen	PRKC, apoptosis, WT1, regulator (PAWR) pseudogene
76	ENSG00000261390	9.11848879	1.39E-17	6.208385228	0.00724341	MAFTRR	16	lincRNA	MAF transcriptional regulator RNA [Source:HGNC Symbol;Acc:HGNC
77	ENSG00000259207	9.11848879	1.39E-17	6.208385228	0.00724341	ITGB3	17	protein_coding	integrin subunit beta 3 [Source:HGNC Symbol;Acc:HGNC:6156]
78	ENSG00000273017	9.11848879	1.39E-17	6.208385228	0.00724341	AP000240.1	21	sense_intronic	novel transcript, sense intronic BACH1
79	ENSG00000213654	43.5619495	13.8186597	1.647445268	0.00725997	GPSM3	6	protein_coding	G protein signaling modulator 3 [Source:HGNC Symbol;Acc:HGNC:1
80	ENSG00000104055	52.6793378	17.7671604	1.561212026	0.00726739	TGM5	15	protein_coding	transglutaminase 5 [Source:HGNC Symbol;Acc:HGNC:11781]

	gene_id	Proglumide	Control	log2FoldChange	pvalue	gene_name	gene_chr	gene_biotype	gene_description
81	ENSG00000105825	54.7054241	18.7542855	1.538061312	0.00753465	TFPI2	7	protein_coding	tissue factor pathway inhibitor 2 [Source:HGNC Symbol;Acc:HGNC:
82	ENSG00000260179	30.3923891	7.89590589	1.927547666	0.00758204	AL162741.1	1	lincRNA	novel transcript
83	ENSG00000198157	15.1967442	1.97313462	2.86753792	0.00786477	HMGN5	X	protein_coding	high mobility group nucleosome binding domain 5 [Source:HGNC S
84	ENSG00000272720	39.509777	11.8444089	1.727244858	0.00794554	AL022322.1	22	lincRNA	novel transcript, antisense to BAIAP2L2
85	ENSG00000025423	25.3271738	5.92165277	2.073258661	0.00813533	HSD17B6	12	protein_coding	hydroxysteroid 17-beta dehydrogenase 6 [Source:HGNC Symbol;Ac
86	ENSG00000114450	12.157616	0.98600522	3.465110713	0.00839028	GNB4	3	protein_coding	G protein subunit beta 4 [Source:HGNC Symbol;Acc:HGNC:20731]
87	ENSG00000196972	12.157616	0.98600522	3.465110713	0.00839028	SMIM10L2B	X	protein_coding	small integral membrane protein 10 like 2B [Source:HGNC Symbol;
88	ENSG00000273599	12.157616	0.98600522	3.465110713	0.00839028	AL731571.1	10	antisense	novel transcript, antisense to CTBP2
89	ENSG00000277011	12.157616	0.98600522	3.465110713	0.00839028	AC148477.4	12	lincRNA	novel transcript
90	ENSG00000197776	12.157616	0.98600522	3.465110713	0.00839028	KLHDC1	14	protein_coding	kelch domain containing 1 [Source:HGNC Symbol;Acc:HGNC:19836
91	ENSG00000158406	20.2619587	3.94739696	2.323231543	0.00845893	HIST1H4H	6	protein_coding	histone cluster 1 H4 family member h [Source:HGNC Symbol;Acc:H
92	ENSG00000230797	20.2619587	3.94739696	2.323231543	0.00845893	YY2	X	protein_coding	YY2 transcription factor [Source:HGNC Symbol;Acc:HGNC:31684]
93	ENSG00000233085	34.4445615	9.87015775	1.789999176	0.00876482	BX322234.2	6	lincRNA	novel transcript
94	ENSG00000223797	53.6923809	18.7542855	1.511156315	0.00878145	ENTPD3-AS1	3	antisense	ENTPD3 antisense RNA 1 [Source:HGNC Symbol;Acc:HGNC:26710]
95	ENSG00000170458	53.6923809	18.7542855	1.511156315	0.00878145	CD14	5	protein_coding	CD14 molecule [Source:HGNC Symbol;Acc:HGNC:1628]
96	ENSG00000215859	48.6271652	16.7800353	1.527888841	0.00970392	PDZK1P1	1	transcribed_unprocess	PDZ domain containing 1 pseudogene 1 [Source:HGNC Symbol;Acc
97	ENSG00000283183	17.2228299	2.96026697	2.490683136	0.01003372	AC116565.1	4	lincRNA	novel transcript
98	ENSG00000261120	17.2228299	2.96026697	2.490683136	0.01003372	AL133299.1	14	antisense	novel transcript, antisense to LRRC9
99	ENSG00000157021	17.2228299	2.96026697	2.490683136	0.01003372	FAM92A1P1	15	unprocessed_pseudog	family with sequence similarity 92 member A1 pseudogene 1 [Sou
100	ENSG00000188243	517.66615	239.87024	1.109355021	0.0100429	COMMD6	13	protein_coding	COMM domain containing 6 [Source:HGNC Symbol;Acc:HGNC:240
101	ENSG00000092607	69.9010713	26.6512854	1.386855781	0.01041416	TBX15	1	protein_coding	T-box 15 [Source:HGNC Symbol;Acc:HGNC:11594]
102	ENSG00000025039	24.3141307	5.92165277	2.014660151	0.01081171	RRAGD	6	protein_coding	Ras related GTP binding D [Source:HGNC Symbol;Acc:HGNC:19903
103	ENSG00000089199	24.3141307	5.92165277	2.014660151	0.01081171	CHGB	20	protein_coding	chromogranin B [Source:HGNC Symbol;Acc:HGNC:1930]
104	ENSG00000280077	43.5619495	14.8057849	1.54877193	0.01085078	AL353763.2	9	TEC	TEC
105	ENSG00000171016	47.6141221	16.7800353	1.497593888	0.01144017	PYGO1	15	protein_coding	pygopus family PHD finger 1 [Source:HGNC Symbol;Acc:HGNC:302
106	ENSG00000236790	14.1837014	1.97313462	2.768842706	0.01183243	LINC00299	2	lincRNA	long intergenic non-protein coding RNA 299 [Source:HGNC Symbol
107	ENSG00000277156	106.370625	45.406658	1.22580339	0.01234179	AL589743.5	14	processed_pseudogen	translocase of outer mitochondrial membrane 40 homolog (yeast)
108	ENSG00000199890	8.10544673	1.39E-17	6.040896957	0.01273085	RF00019	1	misc_RNA	
109	ENSG00000241985	8.10544673	1.39E-17	6.040896957	0.01273085	WWTR1-IT1	3	sense_intronic	WWTR1 intronic transcript 1 [Source:HGNC Symbol;Acc:HGNC:414
110	ENSG00000229619	8.10544673	1.39E-17	6.040896957	0.01273085	MBNL1-AS1	3	antisense	MBNL1 antisense RNA 1 [Source:HGNC Symbol;Acc:HGNC:44584]
111	ENSG00000196542	8.10544673	1.39E-17	6.040896957	0.01273085	SPTSSB	3	protein_coding	serine palmitoyltransferase small subunit B [Source:HGNC Symbol
112	ENSG00000121853	8.10544673	1.39E-17	6.040896957	0.01273085	GHSR	3	protein_coding	growth hormone secretagogue receptor [Source:HGNC Symbol;Acc
113	ENSG00000183171	8.10544673	1.39E-17	6.040896957	0.01273085	BX322650.1	X	processed_pseudogen	nucleolar protein 11, pseudogene
114	ENSG00000120903	8.10544673	1.39E-17	6.040896957	0.01273085	CHRNA2	8	protein_coding	cholinergic receptor nicotinic alpha 2 subunit [Source:HGNC Symb
115	ENSG00000187753	8.10544673	1.39E-17	6.040896957	0.01273085	C9orf153	9	protein_coding	chromosome 9 open reading frame 153 [Source:HGNC Symbol;Acc:
116	ENSG00000244242	8.10544673	1.39E-17	6.040896957	0.01273085	IFITM10	11	protein_coding	interferon induced transmembrane protein 10 [Source:HGNC Symb
117	ENSG00000261594	8.10544673	1.39E-17	6.040896957	0.01273085	TPBGL	11	protein_coding	trophoblast glycoprotein like [Source:HGNC Symbol;Acc:HGNC:441
118	ENSG00000226645	8.10544673	1.39E-17	6.040896957	0.01273085	AP006216.1	11	antisense	novel transcript
119	ENSG00000234859	8.10544673	1.39E-17	6.040896957	0.01273085	AC003958.2	17	antisense	uncharacterized LOC100505782 [Source:NCBI gene;Acc:10050578
120	ENSG00000154262	8.10544673	1.39E-17	6.040896957	0.01273085	ABCA6	17	protein_coding	ATP binding cassette subfamily A member 6 [Source:HGNC Symbol

	gene_id	Proglumide	Control	log2FoldChange	pvalue	gene_name	gene_chr	gene_biotype	gene_description
121	ENSG00000272432	11.1445735	0.98600522	3.340913975	0.01341209	AL031432.3	1	lincRNA	novel transcript
122	ENSG00000280032	11.1445735	0.98600522	3.340913975	0.01341209	AP002800.1	11	TEC	TEC
123	ENSG00000070731	11.1445735	0.98600522	3.340913975	0.01341209	ST6GALNAC2	17	protein_coding	ST6 N-acetylgalactosaminide alpha-2,6-sialyltransferase 2 [Source:
124	ENSG00000242220	11.1445735	0.98600522	3.340913975	0.01341209	TCP10L	21	protein_coding	t-complex 10 like [Source:HGNC Symbol;Acc:HGNC:11657]
125	ENSG00000254860	34.4445615	10.8572834	1.65413649	0.01417872	TMEM9B-AS1	11	antisense	TMEM9B antisense RNA 1 [Source:HGNC Symbol;Acc:HGNC:19230]
126	ENSG00000166343	34.4445615	10.8572834	1.65413649	0.01417872	MSS51	10	protein_coding	MSS51 mitochondrial translational activator [Source:HGNC Symbol;
127	ENSG00000101000	92.1880208	39.4839089	1.220652932	0.01425262	PROCR	20	protein_coding	protein C receptor [Source:HGNC Symbol;Acc:HGNC:9452]
128	ENSG00000270195	23.3010877	5.92165277	1.953580386	0.01438022	AC016773.1	4	lincRNA	novel transcript
129	ENSG00000151846	23.3010877	5.92165277	1.953580386	0.01438022	PABPC3	13	protein_coding	poly(A) binding protein cytoplasmic 3 [Source:HGNC Symbol;Acc:HG
130	ENSG00000138161	16.209787	2.96026697	2.403869845	0.01448543	CUZD1	10	protein_coding	CUB and zona pellucida like domains 1 [Source:HGNC Symbol;Acc:H
131	ENSG00000268621	36.4706477	11.8444089	1.612146733	0.01458372	IGFL2-AS1	19	lincRNA	IGFL2 antisense RNA 1 [Source:HGNC Symbol;Acc:HGNC:52559]
132	ENSG00000269837	112.448884	49.3551573	1.185904831	0.01468926	IPO5P1	19	transcribed_processed	importin 5 pseudogene 1 [Source:HGNC Symbol;Acc:HGNC:49687]
133	ENSG00000264808	27.3532599	7.89590589	1.776200983	0.01605156	AC068025.1	17	antisense	novel transcript, antisense to TAOK1
134	ENSG00000269427	27.3532599	7.89590589	1.776200983	0.01605156	AC024075.3	19	antisense	novel transcript, antisense C19orf42
135	ENSG00000189223	18.2358728	3.94739696	2.172210941	0.01649288	PAX8-AS1	2	processed_transcript	PAX8 antisense RNA 1 [Source:HGNC Symbol;Acc:HGNC:49271]
136	ENSG00000020633	29.379346	8.88303193	1.711416578	0.01665464	RUNX3	1	protein_coding	runt related transcription factor 3 [Source:HGNC Symbol;Acc:HGNC:
137	ENSG00000254231	35.4576046	11.8444089	1.571645529	0.01786044	AC103760.1	8	processed_transcript	uncharacterized LOC105375624 [Source:NCBI gene;Acc:105375624
138	ENSG00000231327	13.1706587	1.97313462	2.662896655	0.01786222	LINC01816	2	lincRNA	long intergenic non-protein coding RNA 1816 [Source:HGNC Symbo
139	ENSG00000177855	13.1706587	1.97313462	2.662896655	0.01786222	CACYBPP2	2	processed_pseudogen	calcyclin binding protein pseudogene 2 [Source:HGNC Symbol;Acc:H
140	ENSG00000269921	13.1706587	1.97313462	2.662896655	0.01786222	AC068620.1	4	lincRNA	novel transcript
141	ENSG00000029534	13.1706587	1.97313462	2.662896655	0.01786222	ANK1	8	protein_coding	ankyrin 1 [Source:HGNC Symbol;Acc:HGNC:492]
142	ENSG00000237082	13.1706587	1.97313462	2.662896655	0.01786222	COX5BP6	13	processed_pseudogen	cytochrome c oxidase subunit 5B pseudogene 6 [Source:HGNC Sym
143	ENSG00000226359	13.1706587	1.97313462	2.662896655	0.01786222	ACTG1P24	17	processed_pseudogen	actin gamma 1 pseudogene 24 [Source:HGNC Symbol;Acc:HGNC:49
144	ENSG00000149599	13.1706587	1.97313462	2.662896655	0.01786222	DUSP15	20	protein_coding	dual specificity phosphatase 15 [Source:HGNC Symbol;Acc:HGNC:1
145	ENSG00000259349	20.2619587	4.93452536	2.010184156	0.01800317	AC011921.1	17	antisense	novel transcript, antisense to APPBP2
146	ENSG00000146674	231.987977	110.556894	1.068387152	0.01801387	IGFBP3	7	protein_coding	insulin like growth factor binding protein 3 [Source:HGNC Symbol;A
147	ENSG00000178809	39.509777	13.8186597	1.507006023	0.01833224	TRIM73	7	protein_coding	tripartite motif containing 73 [Source:HGNC Symbol;Acc:HGNC:181
148	ENSG00000254827	39.509777	13.8186597	1.507006023	0.01833224	SLC22A18AS	11	protein_coding	solute carrier family 22 member 18 antisense [Source:HGNC Symbo
149	ENSG00000019102	41.5358633	14.8057849	1.480260517	0.01850271	VSIG2	11	protein_coding	V-set and immunoglobulin domain containing 2 [Source:HGNC Sym
150	ENSG00000188321	52.6793378	20.7285356	1.340259987	0.01899147	ZNF559	19	protein_coding	zinc finger protein 559 [Source:HGNC Symbol;Acc:HGNC:28197]
151	ENSG00000005513	22.2880447	5.92165277	1.889799908	0.01913737	SOX8	16	protein_coding	SRY-box 8 [Source:HGNC Symbol;Acc:HGNC:11203]
152	ENSG00000128564	135.748877	63.1749046	1.101956778	0.01928491	VGF	7	protein_coding	VGF nerve growth factor inducible [Source:HGNC Symbol;Acc:HGNC
153	ENSG00000172594	177.283646	83.9045253	1.078080754	0.01951749	SMPDL3A	6	protein_coding	sphingomyelin phosphodiesterase acid like 3A [Source:HGNC Symb
154	ENSG00000183087	26.3402168	7.89590589	1.722005585	0.02062376	GAS6	13	protein_coding	growth arrest specific 6 [Source:HGNC Symbol;Acc:HGNC:4168]
155	ENSG00000187566	15.1967442	2.96026697	2.311496245	0.02095211	NHLRC1	6	protein_coding	NHL repeat containing E3 ubiquitin protein ligase 1 [Source:HGNC
156	ENSG00000221947	15.1967442	2.96026697	2.311496245	0.02095211	XKR9	8	protein_coding	XK related 9 [Source:HGNC Symbol;Acc:HGNC:20937]
157	ENSG00000198003	15.1967442	2.96026697	2.311496245	0.02095211	CCDC151	19	protein_coding	coiled-coil domain containing 151 [Source:HGNC Symbol;Acc:HGNC
158	ENSG00000185829	115.488013	53.3036565	1.113575482	0.02120325	ARL17A	17	protein_coding	ADP ribosylation factor like GTPase 17A [Source:HGNC Symbol;Acc:
159	ENSG00000271643	63.8228124	26.6512854	1.255856475	0.02147397	AC112220.2	3	bidirectional_promote	novel transcript
160	ENSG00000231050	10.1315311	0.98600522	3.205009798	0.02156655	AL109917.1	1	antisense	collagen alpha-2(VIII) chain [Source:NCBI gene;Acc:105378949]

	gene_id	Proglumide	Control	log2FoldChange	pvalue	gene_name	gene_chr	gene_biotype	gene_description
160	ENSG00000231050	10.1315311	0.98600522	3.205009798	0.02156655	AL109917.1	1	antisense	collagen alpha-2(VIII) chain [Source:NCBI gene;Acc:105378949]
161	ENSG00000174130	10.1315311	0.98600522	3.205009798	0.02156655	TLR6	4	protein_coding	toll like receptor 6 [Source:HGNC Symbol;Acc:HGNC:16711]
162	ENSG00000272677	10.1315311	0.98600522	3.205009798	0.02156655	AC124016.1	4	antisense	uncharacterized LOC101928963 [Source:NCBI gene;Acc:101928963
163	ENSG00000272218	10.1315311	0.98600522	3.205009798	0.02156655	AC108865.2	4	lincRNA	novel transcript
164	ENSG00000272108	10.1315311	0.98600522	3.205009798	0.02156655	AC244517.1	5	lincRNA	novel transcript
165	ENSG00000131183	10.1315311	0.98600522	3.205009798	0.02156655	SLC34A1	5	protein_coding	solute carrier family 34 member 1 [Source:HGNC Symbol;Acc:HGNC
166	ENSG00000237669	10.1315311	0.98600522	3.205009798	0.02156655	AL671277.2	6	unprocessed_pseudog	HLA complex group 4 pseudogene 3
167	ENSG00000231245	10.1315311	0.98600522	3.205009798	0.02156655	C1DP1	10	processed_pseudogen	C1D nuclear receptor corepressor pseudogene 1 [Source:HGNC Syn
168	ENSG00000246877	10.1315311	0.98600522	3.205009798	0.02156655	DNM1P35	15	antisense	dynamin 1 pseudogene 35 [Source:HGNC Symbol;Acc:HGNC:35182]
169	ENSG00000125409	10.1315311	0.98600522	3.205009798	0.02156655	TEKT3	17	protein_coding	tektin 3 [Source:HGNC Symbol;Acc:HGNC:14293]
170	ENSG00000199961	10.1315311	0.98600522	3.205009798	0.02156655	SNORD1B	17	snoRNA	small nucleolar RNA, C/D box 1B [Source:HGNC Symbol;Acc:HGNC:
171	ENSG00000265496	10.1315311	0.98600522	3.205009798	0.02156655	AC100778.3	18	antisense	microRNA 1539
172	ENSG00000089163	34.4445615	11.8444089	1.5299744	0.02187215	SIRT4	12	protein_coding	sirtuin 4 [Source:HGNC Symbol;Acc:HGNC:14932]
173	ENSG00000159261	36.4706477	12.8315343	1.49782874	0.02199107	CLDN14	21	protein_coding	claudin 14 [Source:HGNC Symbol;Acc:HGNC:2035]
174	ENSG00000223764	7.09240497	1.39E-17	5.851378783	0.02267682	LINC02593	1	lincRNA	long intergenic non-protein coding RNA 2593 [Source:HGNC Symbo
175	ENSG00000235145	7.09240497	1.39E-17	5.851378783	0.02267682	RPSAP16	1	processed_pseudogen	ribosomal protein SA pseudogene 16 [Source:HGNC Symbol;Acc:HG
176	ENSG00000223450	7.09240497	1.39E-17	5.851378783	0.02267682	AL590632.1	1	processed_pseudogen	ribosomal protein S24 (RPS24) pseudogene
177	ENSG00000271868	7.09240497	1.39E-17	5.851378783	0.02267682	AC114810.1	2	lincRNA	novel transcript
178	ENSG00000171303	7.09240497	1.39E-17	5.851378783	0.02267682	KCNK3	2	protein_coding	potassium two pore domain channel subfamily K member 3 [Source
179	ENSG00000258881	7.09240497	1.39E-17	5.851378783	0.02267682	AC007040.2	2	protein_coding	novel protein
180	ENSG00000273113	7.09240497	1.39E-17	5.851378783	0.02267682	AC133528.1	2	lincRNA	novel transcript
181	ENSG00000188996	7.09240497	1.39E-17	5.851378783	0.02267682	HUS1B	6	protein_coding	HUS1 checkpoint clamp component B [Source:HGNC Symbol;Acc:H(
182	ENSG00000220920	7.09240497	1.39E-17	5.851378783	0.02267682	AL023807.1	6	unprocessed_pseudog	ribosomal protein S12 (RPS12) pseudogene
183	ENSG00000124496	7.09240497	1.39E-17	5.851378783	0.02267682	TRERF1	6	protein_coding	transcriptional regulating factor 1 [Source:HGNC Symbol;Acc:HGNC
184	ENSG00000229862	7.09240497	1.39E-17	5.851378783	0.02267682	AL121972.1	6	antisense	novel transcript
185	ENSG00000184530	7.09240497	1.39E-17	5.851378783	0.02267682	C6orf58	6	protein_coding	chromosome 6 open reading frame 58 [Source:HGNC Symbol;Acc:H
186	ENSG00000128606	7.09240497	1.39E-17	5.851378783	0.02267682	LRRC17	7	protein_coding	leucine rich repeat containing 17 [Source:HGNC Symbol;Acc:HGNC:
187	ENSG00000183638	7.09240497	1.39E-17	5.851378783	0.02267682	RP1L1	8	protein_coding	RP1 like 1 [Source:HGNC Symbol;Acc:HGNC:15946]
188	ENSG00000122711	7.09240497	1.39E-17	5.851378783	0.02267682	SPINK4	9	protein_coding	serine peptidase inhibitor, Kazal type 4 [Source:HGNC Symbol;Acc:I
189	ENSG00000165702	7.09240497	1.39E-17	5.851378783	0.02267682	GFI1B	9	protein_coding	growth factor independent 1B transcriptional repressor [Source:HG
190	ENSG00000174885	7.09240497	1.39E-17	5.851378783	0.02267682	NLRP6	11	protein_coding	NLR family pyrin domain containing 6 [Source:HGNC Symbol;Acc:H(
191	ENSG00000254911	7.09240497	1.39E-17	5.851378783	0.02267682	SCARNA9	11	antisense	small Cajal body-specific RNA 9 [Source:HGNC Symbol;Acc:HGNC:3
192	ENSG00000256315	7.09240497	1.39E-17	5.851378783	0.02267682	AP000462.2	11	processed_transcript	uncharacterized LOC105369507 [Source:NCBI gene;Acc:105369507
193	ENSG00000283095	7.09240497	1.39E-17	5.851378783	0.02267682	FP565171.1	10	lincRNA	novel transcript
194	ENSG00000249628	7.09240497	1.39E-17	5.851378783	0.02267682	LINC00942	12	lincRNA	long intergenic non-protein coding RNA 942 [Source:HGNC Symbol;
195	ENSG00000212124	7.09240497	1.39E-17	5.851378783	0.02267682	TAS2R19	12	protein_coding	taste 2 receptor member 19 [Source:HGNC Symbol;Acc:HGNC:1910
196	ENSG00000244712	7.09240497	1.39E-17	5.851378783	0.02267682	AC084754.1	12	processed_pseudogen	ribosomal protein L29 (RPL29) pseudogene
197	ENSG00000139220	7.09240497	1.39E-17	5.851378783	0.02267682	PPFIA2	12	protein_coding	PTPRF interacting protein alpha 2 [Source:HGNC Symbol;Acc:HGNC
198	ENSG00000103313	7.09240497	1.39E-17	5.851378783	0.02267682	MEFV	16	protein_coding	MEFV, pyrin innate immunity regulator [Source:HGNC Symbol;Acc:H
199	ENSG00000266667	7.09240497	1.39E-17	5.851378783	0.02267682	AC005703.4	17	lincRNA	novel transcript
200	ENSG00000266865	7.09240497	1.39E-17	5.851378783	0.02267682	AC138207.8	17	transcribed_unprocess	SMAD specific E3 ubiquitin protein ligase 2 (SMURF2) pseudogene

	gene_id	Proglumide	Control	log2FoldChange	pvalue	gene_name	gene_chr	gene_biotype	gene_description
201	ENSG00000242852	7.09240497	1.39E-17	5.851378783	0.02267682	ZNF709	19	protein_coding	zinc finger protein 709 [Source:HGNC Symbol;Acc:HGNC:20629]
202	ENSG00000254760	7.09240497	1.39E-17	5.851378783	0.02267682	AC008750.1	19	antisense	novel transcript, antisense to SIGLEC10
203	ENSG00000214093	7.09240497	1.39E-17	5.851378783	0.02267682	AL096701.1	22	processed_pseudogen	ribosomal protein S18 (RPS18) pseudogene
204	ENSG00000243811	7.09240497	1.39E-17	5.851378783	0.02267682	APOBEC3D	22	protein_coding	apolipoprotein B mRNA editing enzyme catalytic subunit 3D [Source
205	ENSG00000273254	7.09240497	1.39E-17	5.851378783	0.02267682	AF129075.2	21	antisense	novel transcript, antisense to USP16
206	ENSG00000156265	7.09240497	1.39E-17	5.851378783	0.02267682	MAP3K7CL	21	protein_coding	MAP3K7 C-terminal like [Source:HGNC Symbol;Acc:HGNC:16457]
207	ENSG00000178597	17.2228299	3.94739696	2.090326405	0.02307085	PSAPL1	4	protein_coding	prosaposin like 1 (gene/pseudogene) [Source:HGNC Symbol;Acc:HG
208	ENSG00000196787	17.2228299	3.94739696	2.090326405	0.02307085	HIST1H2AG	6	protein_coding	histone cluster 1 H2A family member g [Source:HGNC Symbol;Acc:H
209	ENSG00000133665	17.2228299	3.94739696	2.090326405	0.02307085	DYDC2	10	protein_coding	DPY30 domain containing 2 [Source:HGNC Symbol;Acc:HGNC:23468
210	ENSG00000262188	17.2228299	3.94739696	2.090326405	0.02307085	LINC01978	17	lincRNA	long intergenic non-protein coding RNA 1978 [Source:HGNC Symbol
211	ENSG00000004766	284.666222	142.144887	1.001257919	0.02308612	VPS50	7	protein_coding	VPS50, EARP/GARPII complex subunit [Source:HGNC Symbol;Acc:HG
212	ENSG00000138772	143.853222	69.0976534	1.05650111	0.02398039	ANXA3	4	protein_coding	annexin A3 [Source:HGNC Symbol;Acc:HGNC:541]
213	ENSG00000100079	115.488013	54.2907814	1.087164634	0.02432645	LGALS2	22	protein_coding	galectin 2 [Source:HGNC Symbol;Acc:HGNC:6562]
214	ENSG00000172167	178.29669	86.8658997	1.036327961	0.02454774	MTBP	8	protein_coding	MDM2 binding protein [Source:HGNC Symbol;Acc:HGNC:7417]
215	ENSG00000138315	80.0315029	36.5225344	1.129041388	0.02523952	OIT3	10	protein_coding	oncoprotein induced transcript 3 [Source:HGNC Symbol;Acc:HGNC:2
216	ENSG00000204272	78.0054165	35.5354095	1.131497308	0.02543552	NBDY	X	protein_coding	negative regulator of P-body association [Source:HGNC Symbol;Acc
217	ENSG00000256591	21.2750017	5.92165277	1.823068794	0.0254753	AP003108.2	11	protein_coding	novel transcript
218	ENSG00000104371	48.6271652	19.7414106	1.295024385	0.02548509	DKK4	8	protein_coding	dickkopf WNT signaling pathway inhibitor 4 [Source:HGNC Symbol;A
219	ENSG00000064886	23.3010877	6.90877955	1.735453749	0.02610533	CHI3L2	1	protein_coding	chitinase 3 like 2 [Source:HGNC Symbol;Acc:HGNC:1933]
220	ENSG00000240602	23.3010877	6.90877955	1.735453749	0.02610533	AADACP1	3	transcribed_unprocess	arylacetamide deacetylase pseudogene 1 [Source:HGNC Symbol;Ac
221	ENSG00000106560	23.3010877	6.90877955	1.735453749	0.02610533	GIMAP2	7	protein_coding	GTPase, IMAP family member 2 [Source:HGNC Symbol;Acc:HGNC:2
222	ENSG00000232807	23.3010877	6.90877955	1.735453749	0.02610533	AL137186.2	10	antisense	uncharacterized LOC105376384 [Source:NCBI gene;Acc:105376384
223	ENSG00000197903	69.9010713	30.5997851	1.188416152	0.02630001	HIST1H2BK	6	protein_coding	histone cluster 1 H2B family member k [Source:HGNC Symbol;Acc:I
224	ENSG00000069812	25.3271738	7.89590589	1.665694583	0.02649769	HES2	1	protein_coding	hes family bHLH transcription factor 2 [Source:HGNC Symbol;Acc:H
225	ENSG00000228705	25.3271738	7.89590589	1.665694583	0.02649769	LINC00659	20	antisense	long intergenic non-protein coding RNA 659 [Source:HGNC Symbol;
226	ENSG00000082196	33.4315184	11.8444089	1.487063742	0.02678003	C1QTNF3	5	protein_coding	C1q and TNF related 3 [Source:HGNC Symbol;Acc:HGNC:14326]
227	ENSG00000145391	29.379346	9.87015775	1.561416423	0.02682007	SETD7	4	protein_coding	SET domain containing lysine methyltransferase 7 [Source:HGNC S
228	ENSG00000184451	29.379346	9.87015775	1.561416423	0.02682007	CCR10	17	protein_coding	C-C motif chemokine receptor 10 [Source:HGNC Symbol;Acc:HGNC:
229	ENSG00000151623	12.157616	1.97313462	2.548549293	0.02704271	NR3C2	4	protein_coding	nuclear receptor subfamily 3 group C member 2 [Source:HGNC Sym
230	ENSG00000231167	12.157616	1.97313462	2.548549293	0.02704271	YBX1P2	7	processed_pseudogen	Y-box binding protein 1 pseudogene 2 [Source:HGNC Symbol;Acc:H
231	ENSG00000285106	12.157616	1.97313462	2.548549293	0.02704271	AC016831.6	7	lincRNA	novel transcript
232	ENSG00000232815	12.157616	1.97313462	2.548549293	0.02704271	DUX4L50	9	unprocessed_pseudog	double homeobox 4 like 50 (pseudogene) [Source:HGNC Symbol;Ac
233	ENSG00000165474	12.157616	1.97313462	2.548549293	0.02704271	GJB2	13	protein_coding	gap junction protein beta 2 [Source:HGNC Symbol;Acc:HGNC:4284]
234	ENSG00000132517	12.157616	1.97313462	2.548549293	0.02704271	SLC52A1	17	protein_coding	solute carrier family 52 member 1 [Source:HGNC Symbol;Acc:HGNC
235	ENSG00000277806	12.157616	1.97313462	2.548549293	0.02704271	AC006213.4	19	sense_intronic	novel transcript, sense intronic to ZNF155
236	ENSG00000236753	117.5141	56.265031	1.060814698	0.02757963	MKLN1-AS	7	processed_transcript	MKLN1 antisense RNA [Source:HGNC Symbol;Acc:HGNC:40374]
237	ENSG00000104427	105.357582	50.3422821	1.063538799	0.02894954	ZC2HC1A	8	protein_coding	zinc finger C2HC-type containing 1A [Source:HGNC Symbol;Acc:HG
238	ENSG00000148908	70.9141145	31.58691	1.163517644	0.028989	RGS10	10	protein_coding	regulator of G protein signaling 10 [Source:HGNC Symbol;Acc:HGN
239	ENSG00000164695	180.322776	89.827274	1.004323733	0.02908983	CHMP4C	8	protein_coding	charged multivesicular body protein 4C [Source:HGNC Symbol;Acc:
240	ENSG00000023445	82.0575892	38.4967841	1.089358586	0.0299605	BIRC3	11	protein_coding	baculoviral IAP repeat containing 3 [Source:HGNC Symbol;Acc:HGN

	gene_id	Proglumide	Control	log2FoldChange	pvalue	gene_name	gene_chr	gene_biotype	gene_description
241	ENSG00000116299	14.1837014	2.96026697	2.212801031	0.03034815	KIAA1324	1	protein_coding	KIAA1324 [Source:HGNC Symbol;Acc:HGNC:29618]
242	ENSG00000280120	40.5228202	15.7929101	1.352390377	0.0311037	AC073857.1	12	TEC	novel transcript
243	ENSG00000096872	144.866265	72.0590278	1.006182082	0.03117997	IFT74	9	protein_coding	intraflagellar transport 74 [Source:HGNC Symbol;Acc:HGNC:21424]
244	ENSG00000234160	58.7575966	25.6641605	1.190989422	0.03121597	AL513165.1	9	antisense	novel transcript
245	ENSG00000167384	102.318452	49.3551573	1.049859035	0.03151617	ZNF180	19	protein_coding	zinc finger protein 180 [Source:HGNC Symbol;Acc:HGNC:12970]
246	ENSG00000213759	38.4967339	14.8057849	1.370977704	0.0315204	UGT2B11	4	protein_coding	UDP glucuronosyltransferase family 2 member B11 [Source:HGNC
247	ENSG00000154016	36.4706477	13.8186597	1.391907899	0.03194235	GRAP	17	protein_coding	GRB2 related adaptor protein [Source:HGNC Symbol;Acc:HGNC:456
248	ENSG00000112406	16.209787	3.94739696	2.003513114	0.03229224	HECA	6	protein_coding	hdc homolog, cell cycle regulator [Source:HGNC Symbol;Acc:HGNC:
249	ENSG00000147003	16.209787	3.94739696	2.003513114	0.03229224	CLTRN	X	protein_coding	collectrin, amino acid transport regulator [Source:HGNC Symbol;Ac
250	ENSG00000282021	16.209787	3.94739696	2.003513114	0.03229224	AC100810.3	8	lincRNA	novel transcript
251	ENSG00000262692	34.4445615	12.8315343	1.415656407	0.03236431	AC116914.2	17	antisense	novel transcript, antisense to ITGAE
252	ENSG00000278611	34.4445615	12.8315343	1.415656407	0.03236431	ZNF426-DT	19	lincRNA	ZNF426 divergent transcript [Source:HGNC Symbol;Acc:HGNC:5377
253	ENSG00000189164	65.8488987	29.6126602	1.149528071	0.0331458	ZNF527	19	protein_coding	zinc finger protein 527 [Source:HGNC Symbol;Acc:HGNC:29385]
254	ENSG00000079557	30.3923891	10.8572834	1.474259593	0.03317368	AFM	4	protein_coding	afamin [Source:HGNC Symbol;Acc:HGNC:316]
255	ENSG00000276538	18.2358728	4.93452536	1.859163554	0.03337634	AC092666.2	7	unprocessed_pseudog	dipeptidyl-peptidase 6 (DPP6) pseudogene
256	ENSG00000257475	18.2358728	4.93452536	1.859163554	0.03337634	AC068888.2	12	sense_intronic	novel transcript
257	ENSG00000225111	28.3663029	9.87015775	1.511008616	0.03353309	AC083900.1	2	sense_intronic	novel transcript
258	ENSG00000255471	20.2619587	5.92165277	1.753100738	0.03391022	AP001528.2	11	antisense	novel transcript
259	ENSG00000281005	20.2619587	5.92165277	1.753100738	0.03391022	LINC00921	16	lincRNA	long intergenic non-protein coding RNA 921 [Source:HGNC Symbol;
260	ENSG00000267508	20.2619587	5.92165277	1.753100738	0.03391022	ZNF285	19	protein_coding	zinc finger protein 285 [Source:HGNC Symbol;Acc:HGNC:13079]
261	ENSG00000253816	24.3141307	7.89590589	1.607096074	0.03403535	AC138866.1	5	unprocessed_pseudog	glucuronidase, beta (GUSB) pseudogene
262	ENSG00000204060	9.11848879	0.98600522	3.054959057	0.0348666	FOXO6	1	protein_coding	forkhead box O6 [Source:HGNC Symbol;Acc:HGNC:24814]
263	ENSG00000240038	9.11848879	0.98600522	3.054959057	0.0348666	AMY2B	1	protein_coding	amylase, alpha 2B (pancreatic) [Source:HGNC Symbol;Acc:HGNC:4
264	ENSG00000248307	9.11848879	0.98600522	3.054959057	0.0348666	LINC00616	4	lincRNA	long intergenic non-protein coding RNA 616 [Source:HGNC Symbo
265	ENSG00000213433	9.11848879	0.98600522	3.054959057	0.0348666	RPLP1P6	5	transcribed_processec	ribosomal protein lateral stalk subunit P1 pseudogene 6 [Source:H
266	ENSG00000179101	9.11848879	0.98600522	3.054959057	0.0348666	AL590139.1	X	processed_pseudogen	H3 histone, family 3B (H3.3B) (H3F3B) pseudogene
267	ENSG00000068976	9.11848879	0.98600522	3.054959057	0.0348666	PYGM	11	protein_coding	glycogen phosphorylase, muscle associated [Source:HGNC Symbo
268	ENSG00000151067	9.11848879	0.98600522	3.054959057	0.0348666	CACNA1C	12	protein_coding	calcium voltage-gated channel subunit alpha1 C [Source:HGNC Sy
269	ENSG00000236617	9.11848879	0.98600522	3.054959057	0.0348666	AC127070.1	12	antisense	uncharacterized LOC101928530 [Source:NCBI gene;Acc:10192853
270	ENSG00000261863	9.11848879	0.98600522	3.054959057	0.0348666	LINC01996	17	lincRNA	long intergenic non-protein coding RNA 1996 [Source:HGNC Symb
271	ENSG00000223573	9.11848879	0.98600522	3.054959057	0.0348666	TINCR	19	protein_coding	TINCR ubiquitin domain containing [Source:HGNC Symbol;Acc:HGI
272	ENSG00000234753	119.540186	59.2264054	1.011610084	0.03504522	FOXP4-AS1	6	antisense	FOXP4 antisense RNA 1 [Source:HGNC Symbol;Acc:HGNC:50332]
273	ENSG00000235689	42.5489064	17.7671604	1.253902591	0.03548196	AC253536.4	22	unprocessed_pseudog	glutathione S-transferase (GST) theta pseudogene
274	ENSG00000119913	80.0315029	38.4967841	1.05334488	0.03607543	TECTB	10	protein_coding	tectorin beta [Source:HGNC Symbol;Acc:HGNC:11721]
275	ENSG00000225335	37.4836908	14.8057849	1.332629742	0.03762082	AC016027.1	22	antisense	novel transcript
276	ENSG00000228889	73.9532439	35.5354095	1.054661786	0.03779259	UBAC2-AS1	13	lincRNA	UBAC2 antisense RNA 1 [Source:HGNC Symbol;Acc:HGNC:42502]
277	ENSG00000260563	73.9532439	35.5354095	1.054661786	0.03779259	AC132872.1	17	lincRNA	novel transcript
278	ENSG00000264920	67.874985	31.58691	1.100437472	0.03981104	AC018521.5	17	processed_transcript	uncharacterized LOC102724532 [Source:NCBI gene;Acc:10272453
279	ENSG00000232560	31.4054322	11.8444089	1.397212591	0.04010141	LINC01549	21	lincRNA	long intergenic non-protein coding RNA 1549 [Source:HGNC Symb
280	ENSG00000116544	6.07936364	1.39E-17	5.633145053	0.04096458	DLGAP3	1	protein_coding	DLG associated protein 3 [Source:HGNC Symbol;Acc:HGNC:30368]

	gene_id	Proglumide	Control	log2FoldChange	pvalue	gene_name	gene_chr	gene_biotype	gene_description
281	ENSG00000234315	6.07936364	1.39E-17	5.633145053	0.04096458	OSTCP5	1	processed_pseudogen	oligosaccharyltransferase complex subunit pseudogene 5 [Source
282	ENSG00000280836	6.07936364	1.39E-17	5.633145053	0.04096458	AL355480.1	1	pseudogene	
283	ENSG00000236915	6.07936364	1.39E-17	5.633145053	0.04096458	AL356270.1	1	antisense	novel transcript, antisense to CLCA4 and CLCA3P
284	ENSG00000225568	6.07936364	1.39E-17	5.633145053	0.04096458	AC093155.1	1	processed_pseudogen	pseudogene similar to part of SUMO-1 activating enzyme subunit
285	ENSG00000228955	6.07936364	1.39E-17	5.633145053	0.04096458	AL591623.2	1	processed_pseudogen	ribosomal protein L7a (RPL7A) pseudogene
286	ENSG00000181378	6.07936364	1.39E-17	5.633145053	0.04096458	CFAP65	2	protein_coding	cilia and flagella associated protein 65 [Source:HGNC Symbol;Acc
287	ENSG00000168427	6.07936364	1.39E-17	5.633145053	0.04096458	KLHL30	2	protein_coding	kelch like family member 30 [Source:HGNC Symbol;Acc:HGNC:247
288	ENSG00000239462	6.07936364	1.39E-17	5.633145053	0.04096458	AC091212.1	3	antisense	uncharacterized LOC105374002 [Source:NCBI gene;Acc:1053740C
289	ENSG00000174500	6.07936364	1.39E-17	5.633145053	0.04096458	GCSAM	3	protein_coding	germinal center associated signaling and motility [Source:HGNC S
290	ENSG00000284624	6.07936364	1.39E-17	5.633145053	0.04096458	AC092902.5	3	processed_transcript	novel transcript
291	ENSG00000250012	6.07936364	1.39E-17	5.633145053	0.04096458	AC079848.1	3	antisense	novel transcript
292	ENSG00000283175	6.07936364	1.39E-17	5.633145053	0.04096458	AC007920.2	3	antisense	uncharacterized LOC101929130 [Source:NCBI gene;Acc:10192913
293	ENSG00000198471	6.07936364	1.39E-17	5.633145053	0.04096458	RTP2	3	protein_coding	receptor transporter protein 2 [Source:HGNC Symbol;Acc:HGNC:32
294	ENSG00000198515	6.07936364	1.39E-17	5.633145053	0.04096458	CNGA1	4	protein_coding	cyclic nucleotide gated channel alpha 1 [Source:HGNC Symbol;Ac
295	ENSG00000245293	6.07936364	1.39E-17	5.633145053	0.04096458	AC096564.1	4	antisense	uncharacterized LOC101929595 [Source:NCBI gene;Acc:10192959
296	ENSG00000249532	6.07936364	1.39E-17	5.633145053	0.04096458	AC106864.1	4	antisense	novel transcript
297	ENSG00000207559	6.07936364	1.39E-17	5.633145053	0.04096458	MIR578	4	miRNA	microRNA 578 [Source:HGNC Symbol;Acc:HGNC:32834]
298	ENSG00000218336	6.07936364	1.39E-17	5.633145053	0.04096458	TENM3	4	protein_coding	teneurin transmembrane protein 3 [Source:HGNC Symbol;Acc:HGI
299	ENSG00000249679	6.07936364	1.39E-17	5.633145053	0.04096458	AC106897.1	4	antisense	uncharacterized LOC105377590 [Source:NCBI gene;Acc:10537759
300	ENSG00000253744	6.07936364	1.39E-17	5.633145053	0.04096458	AC025442.1	5	antisense	novel transcript
301	ENSG00000251467	6.07936364	1.39E-17	5.633145053	0.04096458	AC020893.2	5	processed_pseudogen	dullard homolog (Xenopus laevis) (DULLARD) pseudogene
302	ENSG00000285190	6.07936364	1.39E-17	5.633145053	0.04096458	AC018754.1	5	antisense	novel transcript, antisense to CCNH
303	ENSG00000252464	6.07936364	1.39E-17	5.633145053	0.04096458	RN7SKP70	5	misc_RNA	RNA, 7SK small nuclear pseudogene 70 [Source:HGNC Symbol;Acc:H
304	ENSG00000274997	6.07936364	1.39E-17	5.633145053	0.04096458	HIST1H2AH	6	protein_coding	histone cluster 1 H2A family member h [Source:HGNC Symbol;Acc:H
305	ENSG00000261068	6.07936364	1.39E-17	5.633145053	0.04096458	AL512274.1	6	lincRNA	novel transcript
306	ENSG00000177688	6.07936364	1.39E-17	5.633145053	0.04096458	SUMO4	6	protein_coding	small ubiquitin-like modifier 4 [Source:HGNC Symbol;Acc:HGNC:211
307	ENSG00000230914	6.07936364	1.39E-17	5.633145053	0.04096458	AC004840.1	7	transcribed_unitary_p:	kinesin family member pseudogene
308	ENSG00000253250	6.07936364	1.39E-17	5.633145053	0.04096458	C8orf88	8	protein_coding	chromosome 8 open reading frame 88 [Source:HGNC Symbol;Acc:H
309	ENSG00000279137	6.07936364	1.39E-17	5.633145053	0.04096458	AL356309.3	9	TEC	tec
310	ENSG00000255010	6.07936364	1.39E-17	5.633145053	0.04096458	AP001767.2	11	processed_pseudogen	cytochrome c oxidase subunit Vb (COX5B) pseudogene
311	ENSG00000137673	6.07936364	1.39E-17	5.633145053	0.04096458	MMP7	11	protein_coding	matrix metallopeptidase 7 [Source:HGNC Symbol;Acc:HGNC:7174]
312	ENSG00000255669	6.07936364	1.39E-17	5.633145053	0.04096458	ITFG2-AS1	12	transcribed_unitary_p:	ITFG2 antisense RNA 1 [Source:NCBI gene;Acc:283440]
313	ENSG00000226711	6.07936364	1.39E-17	5.633145053	0.04096458	FAM66C	12	antisense	family with sequence similarity 66 member C [Source:HGNC Symbo
314	ENSG00000259584	6.07936364	1.39E-17	5.633145053	0.04096458	AC021755.2	15	lincRNA	novel transcript
315	ENSG00000259545	6.07936364	1.39E-17	5.633145053	0.04096458	AC013452.1	15	processed_pseudogen	ribosomal protein L15 (RPL15) pseudogene
316	ENSG00000104043	6.07936364	1.39E-17	5.633145053	0.04096458	ATP8B4	15	protein_coding	ATPase phospholipid transporting 8B4 (putative) [Source:HGNC Syn
317	ENSG00000260339	6.07936364	1.39E-17	5.633145053	0.04096458	HEXA-AS1	15	antisense	HEXA antisense RNA 1 [Source:HGNC Symbol;Acc:HGNC:25810]
318	ENSG00000234719	6.07936364	1.39E-17	5.633145053	0.04096458	NPIPB2	16	protein_coding	nuclear pore complex interacting protein family, member B2 [Sourc
319	ENSG00000259940	6.07936364	1.39E-17	5.633145053	0.04096458	AC109449.1	16	antisense	novel transcript, antisense to JMJD5
320	ENSG00000149926	6.07936364	1.39E-17	5.633145053	0.04096458	FAM57B	16	protein_coding	family with sequence similarity 57 member B [Source:HGNC Symbo

	gene_id	Proglumide	Control	log2FoldChange	pvalue	gene_name	gene_chr	gene_biotype	gene_description
321	ENSG00000103089	6.07936364	1.39E-17	5.633145053	0.04096458	FA2H	16	protein_coding	fatty acid 2-hydroxylase [Source:HGNC Symbol;Acc:HGNC:21197]
322	ENSG00000260229	6.07936364	1.39E-17	5.633145053	0.04096458	PPIAP51	16	processed_pseudogen	peptidylprolyl isomerase A pseudogene 51 [Source:HGNC Symbol;Acc
323	ENSG00000131096	6.07936364	1.39E-17	5.633145053	0.04096458	PYY	17	protein_coding	peptide YY [Source:HGNC Symbol;Acc:HGNC:9748]
324	ENSG00000277249	6.07936364	1.39E-17	5.633145053	0.04096458	MIR6784	17	miRNA	microRNA 6784 [Source:HGNC Symbol;Acc:HGNC:49988]
325	ENSG00000272386	6.07936364	1.39E-17	5.633145053	0.04096458	AC015802.5	17	sense_intronic	novel transcript, sense intronic to PRCD
326	ENSG00000196361	6.07936364	1.39E-17	5.633145053	0.04096458	ELAVL3	19	protein_coding	ELAV like RNA binding protein 3 [Source:HGNC Symbol;Acc:HGNC:33
327	ENSG00000267218	6.07936364	1.39E-17	5.633145053	0.04096458	AC005336.2	19	processed_pseudogen	polymerase (RNA) mitochondrial (DNA directed) (POLRMT) pseudoge
328	ENSG00000160460	6.07936364	1.39E-17	5.633145053	0.04096458	SPTBN4	19	protein_coding	spectrin beta, non-erythrocytic 4 [Source:HGNC Symbol;Acc:HGNC:14
329	ENSG00000260101	29.379346	10.8572834	1.425553737	0.04099352	AC008074.2	2	lincRNA	uncharacterized LOC101927402 [Source:NCBI gene;Acc:101927402]
330	ENSG00000224459	11.1445735	1.97313462	2.424352556	0.04103203	SLC25A34-AS	1	antisense	SLC25A34 and TMEM82 antisense RNA 1 [Source:HGNC Symbol;Acc:
331	ENSG00000175920	11.1445735	1.97313462	2.424352556	0.04103203	DOK7	4	protein_coding	docking protein 7 [Source:HGNC Symbol;Acc:HGNC:26594]
332	ENSG00000232946	11.1445735	1.97313462	2.424352556	0.04103203	AL354707.3	9	processed_pseudogen	60S ribosomal protein L35a (RPL35A) pseudogene
333	ENSG00000079337	11.1445735	1.97313462	2.424352556	0.04103203	RAPGEF3	12	protein_coding	Rap guanine nucleotide exchange factor 3 [Source:HGNC Symbol;Acc
334	ENSG00000258900	11.1445735	1.97313462	2.424352556	0.04103203	HNRNPCP1	14	processed_pseudogen	heterogeneous nuclear ribonucleoprotein C pseudogene 1 [Source:H
335	ENSG00000268182	11.1445735	1.97313462	2.424352556	0.04103203	SMIM17	19	protein_coding	small integral membrane protein 17 [Source:HGNC Symbol;Acc:HGNC
336	ENSG00000158246	25.3271738	8.88303193	1.49826935	0.04281416	TENT5B	1	protein_coding	terminal nucleotidyltransferase 5B [Source:HGNC Symbol;Acc:HGNC:
337	ENSG00000162032	25.3271738	8.88303193	1.49826935	0.04281416	SPSB3	16	protein_coding	splA/ryanodine receptor domain and SOCS box containing 3 [Source:
338	ENSG00000178096	87.122805	43.4324083	1.002147005	0.04339667	BOLA1	1	protein_coding	bolA family member 1 [Source:HGNC Symbol;Acc:HGNC:24263]
339	ENSG00000087245	38.4967339	15.7929101	1.278623267	0.04368423	MMP2	16	protein_coding	matrix metallopeptidase 2 [Source:HGNC Symbol;Acc:HGNC:7166]
340	ENSG00000134668	13.1706587	2.96026697	2.106854979	0.04399131	SPOCD1	1	protein_coding	SPOC domain containing 1 [Source:HGNC Symbol;Acc:HGNC:26338]
341	ENSG00000261186	13.1706587	2.96026697	2.106854979	0.04399131	LINC01238	2	antisense	long intergenic non-protein coding RNA 1238 [Source:HGNC Symbo
342	ENSG00000239445	13.1706587	2.96026697	2.106854979	0.04399131	ST3GAL6-AS1	3	antisense	ST3GAL6 antisense RNA 1 [Source:HGNC Symbol;Acc:HGNC:40828]
343	ENSG00000270558	13.1706587	2.96026697	2.106854979	0.04399131	AC025449.1	5	processed_pseudogen	chromobox homolog 3 (CBX3) pseudogene
344	ENSG00000247317	13.1706587	2.96026697	2.106854979	0.04399131	LY6E-DT	8	lincRNA	LY6E divergent transcript [Source:HGNC Symbol;Acc:HGNC:53728]
345	ENSG00000270030	13.1706587	2.96026697	2.106854979	0.04399131	AC136475.7	11	lincRNA	novel transcript
346	ENSG00000272892	13.1706587	2.96026697	2.106854979	0.04399131	AL133551.1	10	lincRNA	novel transcript
347	ENSG00000266200	13.1706587	2.96026697	2.106854979	0.04399131	PNLIPRP2	10	polymorphic_pseudog	pancreatic lipase related protein 2 (gene/pseudogene) [Source:HG
348	ENSG00000102539	13.1706587	2.96026697	2.106854979	0.04399131	MLNR	13	protein_coding	motilin receptor [Source:HGNC Symbol;Acc:HGNC:4495]
349	ENSG00000183921	13.1706587	2.96026697	2.106854979	0.04399131	SDR42E2	16	protein_coding	short chain dehydrogenase/reductase family 42E, member 2 [Sourc
350	ENSG00000280278	13.1706587	2.96026697	2.106854979	0.04399131	FLJ30679	16	TEC	uncharacterized protein FLJ30679 [Source:NCBI gene;Acc:146512]
351	ENSG00000182557	13.1706587	2.96026697	2.106854979	0.04399131	SPNS3	17	protein_coding	sphingolipid transporter 3 (putative) [Source:HGNC Symbol;Acc:HG
352	ENSG00000105419	13.1706587	2.96026697	2.106854979	0.04399131	MEIS3	19	protein_coding	Meis homeobox 3 [Source:HGNC Symbol;Acc:HGNC:29537]
353	ENSG00000281538	13.1706587	2.96026697	2.106854979	0.04399131	AC254562.3	22	lincRNA	novel transcript
354	ENSG00000251495	21.2750017	6.90877955	1.604942157	0.04448909	PPIAP11	5	processed_pseudogen	peptidylprolyl isomerase A pseudogene 11 [Source:HGNC Symbol;A
355	ENSG00000249839	21.2750017	6.90877955	1.604942157	0.04448909	AC011330.1	15	unprocessed_pseudog	histidine acid phosphatase domain containing 2A (HISPPD2A) pseu
356	ENSG00000204103	21.2750017	6.90877955	1.604942157	0.04448909	MAFB	20	protein_coding	MAF bZIP transcription factor B [Source:HGNC Symbol;Acc:HGNC:6
357	ENSG00000163453	55.7184672	25.6641605	1.114535173	0.0451174	IGFBP7	4	protein_coding	insulin like growth factor binding protein 7 [Source:HGNC Symbol;A
358	ENSG00000277476	55.7184672	25.6641605	1.114535173	0.0451174	AC005332.5	17	lincRNA	novel transcript
359	ENSG00000131019	19.2489157	5.92165277	1.679565631	0.04511747	ULBP3	6	protein_coding	UL16 binding protein 3 [Source:HGNC Symbol;Acc:HGNC:14895]
360	ENSG00000230359	19.2489157	5.92165277	1.679565631	0.04511747	TPI1P2	7	transcribed_processe	triosephosphate isomerase 1 pseudogene 2 [Source:HGNC Symbol;

	gene_id	Proglumide	Control	log2FoldChange	pvalue	gene_name	gene_chr	gene_biotype	gene_description
361	ENSG00000104723	19.2489157	5.92165277	1.679565631	0.04511747	TUSC3	8	protein_coding	tumor suppressor candidate 3 [Source:HGNC Symbol;Acc:HGNC:30242
362	ENSG00000232004	19.2489157	5.92165277	1.679565631	0.04511747	CAP1P2	10	processed_pseudogen	CAP1 pseudogene 2 [Source:HGNC Symbol;Acc:HGNC:31970]
363	ENSG00000186642	15.1967442	3.94739696	1.911139514	0.04520218	PDE2A	11	protein_coding	phosphodiesterase 2A [Source:HGNC Symbol;Acc:HGNC:8777]
364	ENSG00000233695	15.1967442	3.94739696	1.911139514	0.04520218	GAS6-AS1	13	antisense	GAS6 antisense RNA 1 [Source:HGNC Symbol;Acc:HGNC:39826]
365	ENSG00000243708	15.1967442	3.94739696	1.911139514	0.04520218	PLA2G4B	15	protein_coding	phospholipase A2 group IVB [Source:HGNC Symbol;Acc:HGNC:9036]
366	ENSG00000250938	17.2228299	4.93452536	1.777279018	0.04543596	AC108866.1	4	antisense	novel transcript, antisense to MAD2L1
367	ENSG00000188199	17.2228299	4.93452536	1.777279018	0.04543596	NUTM2B	10	protein_coding	NUT family member 2B [Source:HGNC Symbol;Acc:HGNC:23445]
368	ENSG00000240291	44.5749927	19.7414106	1.169829721	0.04593126	AL450384.2	10	antisense	novel transcript, antisense to CACNB2
369	ENSG00000227502	67.874985	32.5740349	1.056215668	0.04802793	LINC01268	6	lincRNA	long intergenic non-protein coding RNA 1268 [Source:HGNC Symbol;A
370	ENSG00000247011	30.3923891	11.8444089	1.350097503	0.04902819	AC005920.1	17	lincRNA	novel transcript

Supplemental Table S4: Down- Regulated Genes Control Versus Proglumide

1	gene_id	Proglumide	Control	log2FoldCha	pvalue	gene_name	gene_chr	gene_biotype	gene_description
2	ENSG00000154545	53.6923809	525.149298	-3.2869594	9.91E-12	MAGED4	X	protein_coding	MAGE family member D4 [Source:HGNC Symbol;Acc:HGNC:2
3	ENSG00000205670	6.07936364	96.7371475	-3.9648531	9.72E-10	SMIM11A	21	protein_coding	small integral membrane protein 11A [Source:HGNC Symbol
4	ENSG00000230409	49.6402084	355.363838	-2.8366312	4.06E-09	TCEA1P2	3	processed_pseud	transcription elongation factor A1 pseudogene 2 [Source:HG
5	ENSG00000108821	17.2228299	105.621271	-2.6078863	1.87E-06	COL1A1	17	protein_coding	collagen type I alpha 1 chain [Source:HGNC Symbol;Acc:HGI
6	ENSG00000272320	8.10544673	64.1620294	-2.965704	3.39E-06	AL445309.1	6	antisense	novel transcript, antisense to SLC22A23
7	ENSG00000205572	61.7967261	229.998992	-1.8939262	5.56E-05	SERF1B	5	protein_coding	small EDRK-rich factor 1B [Source:HGNC Symbol;Acc:HGNC:
8	ENSG00000188219	6.07936364	42.4452835	-2.7787872	6.40E-05	POTEE	2	protein_coding	POTE ankyrin domain family member E [Source:HGNC Symb
9	ENSG00000166507	1.01417189	22.7027856	-4.3262449	7.35E-05	NDST2	10	protein_coding	N-deacetylase and N-sulfotransferase 2 [Source:HGNC Syml
10	ENSG00000237399	1.39E-17	17.7671604	-7.1614635	7.55E-05	PITRM1-AS1	10	antisense	PITRM1 antisense RNA 1 [Source:HGNC Symbol;Acc:HGNC:4
11	ENSG00000260075	3.04024516	29.6126602	-3.2324674	0.00011173	NSFP1	17	unprocessed_pse	N-ethylmaleimide-sensitive factor pseudogene 1 [Source:HC
12	ENSG00000174501	32.4184753	124.376641	-1.9357859	0.00011777	ANKRD36C	2	protein_coding	ankyrin repeat domain 36C [Source:HGNC Symbol;Acc:HGNC
13	ENSG00000263961	1.39E-17	16.7800353	-7.079594	0.0001205	RHEX	1	protein_coding	regulator of hemoglobinization and erythroid cell expansion
14	ENSG00000261476	1.39E-17	16.7800353	-7.079594	0.0001205	AC009163.3	16	sense_intronic	novel transcript
15	ENSG00000182463	6.07936364	39.4839089	-2.6747682	0.00013752	TSHZ2	20	protein_coding	teashirt zinc finger homeobox 2 [Source:HGNC Symbol;Acc:H
16	ENSG00000233013	6.07936364	38.4967841	-2.638359	0.00017814	FAM157B	9	transcribed_unpr	family with sequence similarity 157 member B [Source:HGN
17	ENSG00000169894	1.39E-17	15.7929101	-6.9927977	0.00019397	MUC3A	7	protein_coding	mucin 3A, cell surface associated [Source:HGNC Symbol;Acc
18	ENSG00000268521	1.39E-17	15.7929101	-6.9927977	0.00019397	VN1R83P	19	unprocessed_pse	vomeronasal 1 receptor 83 pseudogene [Source:HGNC Symb
19	ENSG00000248318	3.04024516	26.6512854	-3.0811379	0.00029975	AC104958.1	8	antisense	uncharacterized LOC101927543 [Source:NCBI gene;Acc:101
20	ENSG00000255566	1.39E-17	14.8057849	-6.9004432	0.00031521	AC135279.1	12	processed_pseud	mitochondrial ribosomal protein S25 (MRPS25) pseudogene
21	ENSG00000272084	1.39E-17	13.8186597	-6.8017699	0.00051726	AL137127.1	1	3prime_overlappi	novel transcript
22	ENSG00000280392	1.39E-17	13.8186597	-6.8017699	0.00051726	AC007496.3	16	TEC	novel transcript
23	ENSG00000108947	1.39E-17	13.8186597	-6.8017699	0.00051726	EFNB3	17	protein_coding	ephrin B3 [Source:HGNC Symbol;Acc:HGNC:3228]
24	ENSG00000171885	1.39E-17	13.8186597	-6.8017699	0.00051726	AQP4	18	protein_coding	aquaporin 4 [Source:HGNC Symbol;Acc:HGNC:637]
25	ENSG00000268006	9.11848879	44.4195331	-2.2689471	0.00051855	PTOV1-AS1	19	antisense	PTOV1 antisense RNA 1 [Source:HGNC Symbol;Acc:HGNC:44
26	ENSG00000166963	1.01417189	17.7671604	-3.9748004	0.00057541	MAP1A	15	protein_coding	microtubule associated protein 1A [Source:HGNC Symbol;Acc
27	ENSG00000279476	1.01417189	17.7671604	-3.9748004	0.00057541	AC092139.3	16	TEC	novel transcript
28	ENSG00000274292	4.05328324	27.6384104	-2.7326455	0.00063679	AC084018.2	12	lincRNA	novel transcript
29	ENSG00000269821	719.26174	1956.48021	-1.4435162	0.0006398	KCNQ1OT1	11	antisense	KCNQ1 opposite strand/antisense transcript 1 [Source:HGNC
30	ENSG00000230295	9.11848879	42.4452835	-2.203547	0.00064876	GTF2IP23	7	unprocessed_pse	general transcription factor IIi pseudogene 23 [Source:HGNC
31	ENSG00000264268	2.02720959	20.7285356	-3.277236	0.00073951	MIR4767	X	miRNA	microRNA 4767 [Source:HGNC Symbol;Acc:HGNC:41548]
32	ENSG00000268189	2.02720959	20.7285356	-3.277236	0.00073951	AC005785.1	19	processed_transc	novel transcript, antisense to AKAP8 and AKAP8L
33	ENSG00000274641	1.39E-17	12.8315343	-6.695849	0.00085757	HIST1H2BO	6	protein_coding	histone cluster 1 H2B family member o [Source:HGNC Symb
34	ENSG00000106031	1.39E-17	12.8315343	-6.695849	0.00085757	HOXA13	7	protein_coding	homeobox A13 [Source:HGNC Symbol;Acc:HGNC:5102]
35	ENSG00000145002	1.39E-17	12.8315343	-6.695849	0.00085757	FAM86B2	8	protein_coding	family with sequence similarity 86 member B2 [Source:HGN
36	ENSG00000254013	1.39E-17	12.8315343	-6.695849	0.00085757	MAP2K1P1	8	processed_pseud	mitogen-activated protein kinase kinase 1 pseudogene 1 [Sc
37	ENSG00000226981	1.39E-17	12.8315343	-6.695849	0.00085757	ABHD17AP6	17	unprocessed_pse	abhydrolase domain containing 17A pseudogene 6 [Source:H
38	ENSG00000209042	1.39E-17	12.8315343	-6.695849	0.00085757	SNORD12C	20	snoRNA	small nucleolar RNA, C/D box 12C [Source:HGNC Symbol;Acc
39	ENSG00000236264	7.09240497	35.5354095	-2.3050371	0.00086093	RPL26P30	11	transcribed_proc	ribosomal protein L26 pseudogene 30 [Source:HGNC Symbol
40	ENSG00000218537	4.05328324	26.6512854	-2.6804186	0.00087204	MIF-AS1	22	antisense	MIF antisense RNA 1 [Source:HGNC Symbol;Acc:HGNC:2766

41	ENSG00000233654	1.01417189	16.7800353	-3.8929309	0.00088486	AC108047.1	2	antisense	novel transcript
42	ENSG00000142798	3360.26527	8759.74412	-1.3822812	0.00095983	HSPG2	1	protein_coding	heparan sulfate proteoglycan 2 [Source:HGNC Symbol;Acc:H
43	ENSG00000230953	2.02720959	19.7414106	-3.207279	0.00108459	AC099677.1	1	processed_pseud	translational machinery associated 7 homolog (S. cerevisiae
44	ENSG00000255139	2.02720959	19.7414106	-3.207279	0.00108459	AP000442.2	11	antisense	novel transcript
45	ENSG00000167548	1620.87016	4182.44656	-1.3675118	0.00111441	KMT2D	12	protein_coding	lysine methyltransferase 2D [Source:HGNC Symbol;Acc:HGN
46	ENSG00000231226	6.07936364	31.58691	-2.3539777	0.00115274	TRIM31-AS1	6	antisense	TRIM31 antisense RNA 1 [Source:HGNC Symbol;Acc:HGNC:3
47	ENSG00000257315	3.04024516	22.7027856	-2.850983	0.0011736	ZBED6	1	protein_coding	zinc finger BED-type containing 6 [Source:HGNC Symbol;Acc
48	ENSG00000186364	24.3141307	79.9560261	-1.712352	0.00124079	NUDT17	1	protein_coding	nudix hydrolase 17 [Source:HGNC Symbol;Acc:HGNC:26618]
49	ENSG00000252743	1.01417189	15.7929101	-3.8061345	0.00136958	RNU6-850P	6	snRNA	RNA, U6 small nuclear 850, pseudogene [Source:HGNC Sym
50	ENSG00000279841	1.01417189	15.7929101	-3.8061345	0.00136958	AC092135.3	16	TEC	novel transcript
51	ENSG00000279652	1.01417189	15.7929101	-3.8061345	0.00136958	Z82217.1	22	lincRNA	novel transcript
52	ENSG00000259366	11.1445735	45.406658	-2.014613	0.00140429	AC108449.2	8	sense_intronic	novel transcript, sense intronic to HMBOX1
53	ENSG00000280213	33.4315184	101.672771	-1.6011037	0.00142274	UCKL1-AS1	20	antisense	UCKL1 antisense RNA 1 [Source:HGNC Symbol;Acc:HGNC:31
54	ENSG00000264349	1.39E-17	11.8444089	-6.581531	0.00143701	MIR4258	1	miRNA	microRNA 4258 [Source:HGNC Symbol;Acc:HGNC:38281]
55	ENSG00000232713	1.39E-17	11.8444089	-6.581531	0.00143701	AC010733.1	2	processed_pseud	ribosomal protein S12 (RPS12) pseudogene
56	ENSG00000231760	1.39E-17	11.8444089	-6.581531	0.00143701	AL355312.2	6	antisense	novel transcript
57	ENSG00000275097	1.39E-17	11.8444089	-6.581531	0.00143701	AC024940.5	12	3prime_overlappi	novel transcript, overlapping 3'UTR of FAM60A
58	ENSG00000259209	1.39E-17	11.8444089	-6.581531	0.00143701	AC004943.1	16	antisense	novel transcript, antisense to ZFHX3
59	ENSG00000158270	1.39E-17	11.8444089	-6.581531	0.00143701	COLEC12	18	protein_coding	collectin subfamily member 12 [Source:HGNC Symbol;Acc:H
60	ENSG00000089847	1.39E-17	11.8444089	-6.581531	0.00143701	ANKRD24	19	protein_coding	ankyrin repeat domain 24 [Source:HGNC Symbol;Acc:HGNC:
61	ENSG00000254413	1.39E-17	11.8444089	-6.581531	0.00143701	CHKB-CPT1B	22	protein_coding	CHKB-CPT1B readthrough (NMD candidate) [Source:HGNC S
62	ENSG00000234030	1.39E-17	11.8444089	-6.581531	0.00143701	TMEM97P1	21	processed_pseud	transmembrane protein 97 pseudogene 1 [Source:HGNC Sym
63	ENSG00000232630	4.05328324	24.6770356	-2.5699259	0.0016487	PRPS1P2	9	processed_pseud	phosphoribosyl pyrophosphate synthetase 1 pseudogene 2 [
64	ENSG00000142512	3.04024516	21.7156606	-2.7872117	0.00166513	SIGLEC10	19	protein_coding	sialic acid binding Ig like lectin 10 [Source:HGNC Symbol;Acc
65	ENSG00000235499	8.10544673	35.5354095	-2.1155189	0.00172833	AC073046.1	2	lincRNA	novel transcript (LOC100132318)
66	ENSG00000168016	12.157616	46.3937828	-1.9213589	0.00195502	TRANK1	3	protein_coding	tetratricopeptide repeat and ankyrin repeat containing 1 [So
67	ENSG00000263968	6.07936364	29.6126602	-2.2612473	0.00199893	RN7SL381P	16	misc_RNA	RNA, 7SL, cytoplasmic 381, pseudogene [Source:HGNC Symb
68	ENSG00000257989	1.01417189	14.8057849	-3.7137801	0.00213372	AC078864.1	12	lincRNA	novel transcript
69	ENSG00000233622	1.01417189	14.8057849	-3.7137801	0.00213372	CYP2T1P	19	transcribed_unita	cytochrome P450 family 2 subfamily T member 1, pseudoger
70	ENSG00000203761	57.7445535	158.926009	-1.4586503	0.00217669	MSTO2P	1	unprocessed_pse	misato family member 2, pseudogene [Source:HGNC Symbol
71	ENSG00000273295	13.1706587	48.3680324	-1.8669743	0.00218391	AP000350.6	22	lincRNA	novel transcript
72	ENSG00000230021	4.05328324	23.6899106	-2.5113351	0.00227587	AL669831.3	1	transcribed_proc	uncharacterized LOC101928626 [Source:NCBI gene;Acc:1019
73	ENSG00000224751	1.39E-17	10.8572834	-6.457369	0.00243493	SHMT1P1	1	processed_pseud	serine hydroxymethyltransferase 1 (soluble) pseudogene 1 [
74	ENSG00000251634	1.39E-17	10.8572834	-6.457369	0.00243493	AC145138.1	5	unprocessed_pse	NLR family, apoptosis inhibitory protein (NAIP) pseudogene
75	ENSG00000201785	1.39E-17	10.8572834	-6.457369	0.00243493	SNORD117	6	snoRNA	small nucleolar RNA, C/D box 117 [Source:HGNC Symbol;Acc
76	ENSG00000231519	1.39E-17	10.8572834	-6.457369	0.00243493	AC007285.2	7	antisense	novel transcript
77	ENSG00000227329	1.39E-17	10.8572834	-6.457369	0.00243493	AL139396.1	X	processed_pseud	pseudogene similar to part of suppressor of variegation 3-9
78	ENSG00000255236	1.39E-17	10.8572834	-6.457369	0.00243493	AP002992.1	11	antisense	novel transcript
79	ENSG00000149380	1.39E-17	10.8572834	-6.457369	0.00243493	P4HA3	11	protein_coding	prolyl 4-hydroxylase subunit alpha 3 [Source:HGNC Symbol;A
80	ENSG00000185567	9.11848879	36.5225344	-1.9874223	0.00255104	AHNAK2	14	protein_coding	AHNAK nucleoprotein 2 [Source:HGNC Symbol;Acc:HGNC:20

81	ENSG00000226450	11.1445735	41.4581586	-1.8837456	0.00261795	CYP2D8P	22	unprocessed_pse	cytochrome P450 family 2 subfamily D member 8, pseudog
82	ENSG00000237719	6.07936364	28.6255353	-2.2125468	0.002639	Z95152.1	6	processed_pseuc	nudix (nucleoside diphosphate linked moiety X) type 5 motif
83	ENSG00000260884	6.07936364	28.6255353	-2.2125468	0.002639	AC009120.3	16	lincRNA	novel transcript
84	ENSG00000142178	67.874985	178.668504	-1.39472	0.00279882	SIK1	21	protein_coding	salt inducible kinase 1 [Source:HGNC Symbol;Acc:HGNC:111
85	ENSG00000218226	103.331496	259.612735	-1.3280527	0.00305522	TATDN2P2	6	processed_pseuc	TatD DNase domain containing 2 pseudogene 2 [Source:HGI
86	ENSG00000133069	4.05328324	22.7027856	-2.4502637	0.00314942	TMCC2	1	protein_coding	transmembrane and coiled-coil domain family 2 [Source:HGI
87	ENSG00000239704	4.05328324	22.7027856	-2.4502637	0.00314942	CDRT4	17	protein_coding	CMT1A duplicated region transcript 4 [Source:HGNC Symbol;
88	ENSG00000227388	9.11848879	35.5354095	-1.9480306	0.00321704	AL133410.1	9	lincRNA	novel transcript
89	ENSG00000106823	1.01417189	13.8186597	-3.6151068	0.00334606	ECM2	9	protein_coding	extracellular matrix protein 2 [Source:HGNC Symbol;Acc:HGI
90	ENSG00000167618	1.01417189	13.8186597	-3.6151068	0.00334606	LAIR2	19	protein_coding	leukocyte associated immunoglobulin like receptor 2 [Source
91	ENSG00000261884	6.07936364	27.6384104	-2.1621447	0.00348955	AC040162.1	16	protein_coding	novel transcript
92	ENSG00000187987	2.02720959	16.7800353	-2.9744145	0.00351516	ZSCAN23	6	protein_coding	zinc finger and SCAN domain containing 23 [Source:HGNC Sy
93	ENSG00000235290	2.02720959	16.7800353	-2.9744145	0.00351516	HLA-W	6	unprocessed_pse	major histocompatibility complex, class I, W (pseudogene) [
94	ENSG00000092200	2.02720959	16.7800353	-2.9744145	0.00351516	RPGRIP1	14	protein_coding	RPGR interacting protein 1 [Source:HGNC Symbol;Acc:HGNC:
95	ENSG00000276317	2.02720959	16.7800353	-2.9744145	0.00351516	AL357033.3	20	antisense	novel transcript, antisense to SLCO4A1
96	ENSG00000236761	1.39E-17	9.87015775	-6.3215063	0.00417417	CTAGE9	6	protein_coding	CTAGE family member 9 [Source:HGNC Symbol;Acc:HGNC:37
97	ENSG00000237243	1.39E-17	9.87015775	-6.3215063	0.00417417	AC022173.1	7	lincRNA	uncharacterized LOC100130880 [Source:NCBI gene;Acc:1001
98	ENSG00000186471	1.39E-17	9.87015775	-6.3215063	0.00417417	AKAP14	X	protein_coding	A-kinase anchoring protein 14 [Source:HGNC Symbol;Acc:HG
99	ENSG00000235961	1.39E-17	9.87015775	-6.3215063	0.00417417	PNMA6A	X	protein_coding	PNMA family member 6A [Source:HGNC Symbol;Acc:HGNC:2
100	ENSG00000253586	1.39E-17	9.87015775	-6.3215063	0.00417417	AC067817.1	8	sense_intronic	novel transcript
101	ENSG00000259121	1.39E-17	9.87015775	-6.3215063	0.00417417	AL109628.1	14	processed_pseuc	ribosomal protein, large, P1 (RPLP1) pseudogene
102	ENSG00000253674	1.39E-17	9.87015775	-6.3215063	0.00417417	IGHVII-78-1	14	IG_V_pseudogen	immunoglobulin heavy variable (II)-78-1 (pseudogene) [Sourc
103	ENSG00000264937	1.39E-17	9.87015775	-6.3215063	0.00417417	AC100830.2	15	antisense	novel transcript, antisense to ornithine decarboxylase antizyr
104	ENSG00000260874	1.39E-17	9.87015775	-6.3215063	0.00417417	AC106820.5	16	lincRNA	uncharacterized LOC105371050 [Source:NCBI gene;Acc:1053
105	ENSG00000266076	1.39E-17	9.87015775	-6.3215063	0.00417417	AC004805.1	17	protein_coding	novel transcript
106	ENSG00000270993	1.39E-17	9.87015775	-6.3215063	0.00417417	AC111186.1	17	processed_pseuc	WD repeat domain, phosphoinositide interacting 2 (WIPI2) ps
107	ENSG00000267908	1.39E-17	9.87015775	-6.3215063	0.00417417	ZSCAN5DP	19	unprocessed_pse	zinc finger and SCAN domain containing 5D pseudogene [Sou
108	ENSG00000273343	4.05328324	21.7156606	-2.3864924	0.00436855	AC007663.4	22	sense_intronic	novel transcript, sense intronic to DGCR6L
109	ENSG00000155657	56.7315104	146.093386	-1.3627658	0.00441944	TTN	2	protein_coding	titin [Source:HGNC Symbol;Acc:HGNC:12403]
110	ENSG00000167244	3189.06098	7231.67497	-1.1811673	0.0045965	IGF2	11	protein_coding	insulin like growth factor 2 [Source:HGNC Symbol;Acc:HGNC:
111	ENSG00000283689	8.10544673	31.58691	-1.9462258	0.00461337	AC018553.2	16	antisense	novel transcript, antisense to IRX3
112	ENSG00000065675	3.04024516	18.7542855	-2.5770105	0.00484911	PRKCQ	10	protein_coding	protein kinase C theta [Source:HGNC Symbol;Acc:HGNC:9410
113	ENSG00000263096	43.5619495	114.505394	-1.3917652	0.00490444	AC007638.2	17	antisense	novel transcript, antisense to HLF
114	ENSG00000224186	30.3923891	82.9174005	-1.4442874	0.00497182	C5orf66	5	antisense	chromosome 5 open reading frame 66 [Source:HGNC Symbol;
115	ENSG00000197301	17.2228299	53.3036565	-1.6229795	0.00503207	AC090673.1	12	antisense	uncharacterized LOC100129940 [Source:NCBI gene;Acc:1001
116	ENSG00000230424	2.02720959	15.7929101	-2.8876181	0.00524731	AL035413.1	1	antisense	uncharacterized LOC101927895 [Source:NCBI gene;Acc:1019
117	ENSG00000262180	2.02720959	15.7929101	-2.8876181	0.00524731	OCLM	1	protein_coding	oculomedin [Source:HGNC Symbol;Acc:HGNC:8103]
118	ENSG00000183943	2.02720959	15.7929101	-2.8876181	0.00524731	PRKX	X	protein_coding	protein kinase X-linked [Source:HGNC Symbol;Acc:HGNC:944
119	ENSG00000266777	2.02720959	15.7929101	-2.8876181	0.00524731	SH3GL1P1	17	transcribed_proc	SH3 domain containing GRB2 like 1, endophilin A2 pseudoge
120	ENSG00000231119	2.02720959	15.7929101	-2.8876181	0.00524731	AL031666.1	20	antisense	uncharacterized LOC101927377 [Source:NCBI gene;Acc:1019

121	ENSG00000231467	2.02720959	15.7929101	-2.8876181	0.00524731	AL022314.1	22	antisense	novel transcript
122	ENSG00000137261	1.01417189	12.8315343	-3.5091859	0.00528154	KIAA0319	6	protein_coding	KIAA0319 [Source:HGNC Symbol;Acc:HGNC:21580]
123	ENSG00000228327	55.7184672	141.157763	-1.3391645	0.00532101	AL669831.1	1	transcribed_unpr	general transcription factor IIi (GTF2I) pseudogene
124	ENSG00000159917	15.1967442	48.3680324	-1.662333	0.00533667	ZNF235	19	protein_coding	zinc finger protein 235 [Source:HGNC Symbol;Acc:HGNC:128
125	ENSG00000084674	65284.5549	144767.769	-1.1489253	0.00568134	APOB	2	protein_coding	apolipoprotein B [Source:HGNC Symbol;Acc:HGNC:603]
126	ENSG00000272556	95.2271502	226.050493	-1.2461287	0.00589811	GTF2IP13	7	transcribed_unpr	general transcription factor IIi pseudogene 13 [Source:HGNC
127	ENSG00000110025	11.1445735	37.5096592	-1.7398121	0.00606867	SNX15	11	protein_coding	sorting nexin 15 [Source:HGNC Symbol;Acc:HGNC:14978]
128	ENSG00000235408	6.07936364	25.6641605	-2.055729	0.00612771	SNORA71B	20	snoRNA	small nucleolar RNA, H/ACA box 71B [Source:HGNC Symbol;.
129	ENSG00000232034	9.11848879	32.5740349	-1.8229594	0.00648527	AC092168.2	2	sense_intronic	novel transcript
130	ENSG00000279662	30.3923891	79.9560261	-1.3919004	0.00694615	AC131649.2	16	TEC	TEC
131	ENSG00000229178	3.04024516	17.7671604	-2.4995385	0.00696616	AC233280.1	3	lincRNA	novel transcript
132	ENSG00000182175	3.04024516	17.7671604	-2.4995385	0.00696616	RGMA	15	protein_coding	repulsive guidance molecule BMP co-receptor a [Source:HGI
133	ENSG00000226763	3.04024516	17.7671604	-2.4995385	0.00696616	SRRM5	19	protein_coding	serine/arginine repetitive matrix 5 [Source:HGNC Symbol;Ac
134	ENSG00000236364	5.06632294	22.7027856	-2.1369952	0.00716778	AL358115.1	1	antisense	novel transcript, antisense to UCK2
135	ENSG00000207805	5.06632294	22.7027856	-2.1369952	0.00716778	MIR483	11	miRNA	microRNA 483 [Source:HGNC Symbol;Acc:HGNC:32340]
136	ENSG00000215493	5.06632294	22.7027856	-2.1369952	0.00716778	AC007731.2	22	processed_pseud	kelch-like 12 (Drosophila) (KLH12) pseudogene
137	ENSG00000235831	1.39E-17	8.88303193	-6.1715061	0.00724341	BHLHE40-AS1	3	antisense	BHLHE40 antisense RNA 1 [Source:HGNC Symbol;Acc:HGNC:
138	ENSG00000253119	1.39E-17	8.88303193	-6.1715061	0.00724341	HNRNPA3P7	5	processed_pseud	heterogeneous nuclear ribonucleoprotein A3 pseudogene 7 [
139	ENSG00000237065	1.39E-17	8.88303193	-6.1715061	0.00724341	NANOGP4	7	transcribed_proc	Nanog homeobox pseudogene 4 [Source:HGNC Symbol;Acc:H
140	ENSG00000123965	1.39E-17	8.88303193	-6.1715061	0.00724341	PMS2P5	7	unprocessed_pse	PMS1 homolog 2, mismatch repair system component pseud
141	ENSG00000243566	1.39E-17	8.88303193	-6.1715061	0.00724341	UPK3B	7	protein_coding	uroplakin 3B [Source:HGNC Symbol;Acc:HGNC:21444]
142	ENSG00000208036	1.39E-17	8.88303193	-6.1715061	0.00724341	MIR106B	7	miRNA	microRNA 106b [Source:HGNC Symbol;Acc:HGNC:31495]
143	ENSG00000282977	1.39E-17	8.88303193	-6.1715061	0.00724341	PCBP2-OT1	12	non_coding	PCBP2 overlapping transcript 1 [Source:HGNC Symbol;Acc:H
144	ENSG00000236526	1.39E-17	8.88303193	-6.1715061	0.00724341	AL035448.1	20	antisense	novel transcript
145	ENSG00000093100	1.39E-17	8.88303193	-6.1715061	0.00724341	AC016026.1	22	processed_transc	novel transcript
146	ENSG00000005810	286.692308	635.707272	-1.1485221	0.00740589	MYCBP2	13	protein_coding	MYC binding protein 2, E3 ubiquitin protein ligase [Source:H
147	ENSG00000038219	305.940128	677.166513	-1.1459457	0.00741305	BOD1L1	4	protein_coding	biorientation of chromosomes in cell division 1 like 1 [Sourc
148	ENSG00000116127	185.387992	414.591324	-1.1606169	0.00749452	ALMS1	2	protein_coding	ALMS1, centrosome and basal body associated protein [Sou
149	ENSG00000223901	19.2489157	55.2779062	-1.5159563	0.00753465	AP001469.1	21	antisense	novel transcript
150	ENSG00000247853	2.02720959	14.8057849	-2.7952637	0.00786477	AC006064.2	12	antisense	novel transcript, antisense to CHD4
151	ENSG00000257896	2.02720959	14.8057849	-2.7952637	0.00786477	AC093012.1	12	transcribed_unpr	La ribonucleoprotein domain family, member 7 (LARP7)
152	ENSG00000159337	2.02720959	14.8057849	-2.7952637	0.00786477	PLA2G4D	15	protein_coding	phospholipase A2 group IVD [Source:HGNC Symbol;Acc:HGN
153	ENSG00000232645	2.02720959	14.8057849	-2.7952637	0.00786477	LINC01431	20	lincRNA	long intergenic non-protein coding RNA 1431 [Source:HGNC
154	ENSG00000276570	12.157616	38.4967841	-1.6529672	0.00794554	AC010327.5	19	processed_transc	novel transcript
155	ENSG00000030419	6.07936364	24.6770356	-1.9994251	0.00813533	IKZF2	2	protein_coding	IKAROS family zinc finger 2 [Source:HGNC Symbol;Acc:HGN(
156	ENSG00000279641	6.07936364	24.6770356	-1.9994251	0.00813533	AC120057.3	17	TEC	TEC
157	ENSG00000204778	13.1706587	40.4710338	-1.6105418	0.00835646	CBWD4P	9	unprocessed_pse	COBW domain containing 4 pseudogene [Source:HGNC Sym
158	ENSG00000263069	13.1706587	40.4710338	-1.6105418	0.00835646	AC124319.2	17	antisense	uncharacterized LOC100294362 [Source:NCBI gene;Acc:100
159	ENSG00000146216	28.3663029	74.0332774	-1.3801629	0.0083568	TTBK1	6	protein_coding	tau tubulin kinase 1 [Source:HGNC Symbol;Acc:HGNC:19140
160	ENSG00000248641	1.01417189	11.8444089	-3.3948679	0.00839028	HMGA1P2	4	processed_pseud	high mobility group AT-hook 1 pseudogene 2 [Source:HGNC

161	ENSG00000249655	1.01417189	11.8444089	-3.3948679	0.00839028	AC008434.1	5	antisense	novel transcript
162	ENSG00000251728	1.01417189	11.8444089	-3.3948679	0.00839028	RF00019	7	misc_RNA	
163	ENSG00000107249	1.01417189	11.8444089	-3.3948679	0.00839028	GLIS3	9	protein_coding	GLIS family zinc finger 3 [Source:HGNC Symbol;Acc:HGNC:28
164	ENSG00000160408	1.01417189	11.8444089	-3.3948679	0.00839028	ST6GALNAC6	9	protein_coding	ST6 N-acetylgalactosaminide alpha-2,6-sialyltransferase 6 [
165	ENSG00000260394	1.01417189	11.8444089	-3.3948679	0.00839028	Z92544.1	16	antisense	uncharacterized LOC105371184 [Source:NCBI gene;Acc:105
166	ENSG00000278986	1.01417189	11.8444089	-3.3948679	0.00839028	AC091060.1	18	TEC	TEC
167	ENSG00000269172	1.01417189	11.8444089	-3.3948679	0.00839028	AC011443.1	19	antisense	novel transcript, antisense to PAK4
168	ENSG00000133454	1.01417189	11.8444089	-3.3948679	0.00839028	MYO18B	22	protein_coding	myosin XVIIIB [Source:HGNC Symbol;Acc:HGNC:18150]
169	ENSG00000176542	208.687984	460.986188	-1.1429142	0.00847823	USF3	3	protein_coding	upstream transcription factor family member 3 [Source:HGN
170	ENSG00000179899	24.3141307	65.1491542	-1.4174078	0.00867488	PHC1P1	12	processed_pseud	polyhomeotic homolog 1 pseudogene 1 [Source:HGNC Symb
171	ENSG00000066056	25.3271738	67.1234038	-1.4017967	0.00886945	TIE1	1	protein_coding	tyrosine kinase with immunoglobulin like and EGF like doma
172	ENSG00000156804	7.09240497	26.6512854	-1.8916841	0.00898543	FBXO32	8	protein_coding	F-box protein 32 [Source:HGNC Symbol;Acc:HGNC:16731]
173	ENSG00000183458	405.218359	871.630092	-1.1047841	0.00916938	AC138932.1	16	transcribed_unpr	polycystic kidney disease 1 (autosomal dominant) (PKD1) ps
174	ENSG00000231073	8.10544673	28.6255353	-1.8047949	0.00973082	AL590133.1	1	antisense	novel transcript, antisense to CERS2
175	ENSG00000227934	5.06632294	21.7156606	-2.0732239	0.00973269	AL732414.1	1	processed_pseud	ring finger protein 4 (RNF4) pseudogene
176	ENSG00000224975	5.06632294	21.7156606	-2.0732239	0.00973269	INE1	X	sense_intronic	inactivation escape 1 [Source:HGNC Symbol;Acc:HGNC:6060
177	ENSG00000167889	5.06632294	21.7156606	-2.0732239	0.00973269	MGAT5B	17	protein_coding	alpha-1,6-mannosylglycoprotein 6-beta-N-acetylglucosamin
178	ENSG00000148773	1678.61362	3541.80259	-1.0771585	0.00978725	MKI67	10	protein_coding	marker of proliferation Ki-67 [Source:HGNC Symbol;Acc:HGN
179	ENSG00000197714	85.0967186	192.488251	-1.1764406	0.00993505	ZNF460	19	protein_coding	zinc finger protein 460 [Source:HGNC Symbol;Acc:HGNC:216
180	ENSG00000230732	3.04024516	16.7800353	-2.417669	0.01003372	AC016949.1	3	sense_intronic	novel transcript
181	ENSG00000128918	3.04024516	16.7800353	-2.417669	0.01003372	ALDH1A2	15	protein_coding	aldehyde dehydrogenase 1 family member A2 [Source:H
182	ENSG00000272115	13.1706587	39.4839089	-1.575029	0.01013842	AC233992.3	8	antisense	novel transcript, antisense to SLC39A4
183	ENSG00000271550	6.07936364	23.6899106	-1.9408343	0.01081171	BNIP3P11	7	processed_pseud	BCL2 interacting protein 3 pseudogene 11 [Source:HGN
184	ENSG00000054654	144.866265	314.891722	-1.1194783	0.01123117	SYNE2	14	protein_coding	spectrin repeat containing nuclear envelope protein 2 [
185	ENSG00000103647	24.3141307	63.1749046	-1.3731	0.0112742	CORO2B	15	protein_coding	coronin 2B [Source:HGNC Symbol;Acc:HGNC:2256]
186	ENSG00000163884	67.874985	154.977509	-1.189649	0.01129721	KLF15	3	protein_coding	Kruppel like factor 15 [Source:HGNC Symbol;Acc:HGNC:
187	ENSG00000053438	18.2358728	50.3422821	-1.4588313	0.01169695	NNAT	20	protein_coding	neuronatin [Source:HGNC Symbol;Acc:HGNC:7860]
188	ENSG00000272655	7.09240497	25.6641605	-1.8374953	0.01172386	POLR2J4	7	transcribed_unpr	RNA polymerase II subunit J4, pseudogene [Source:HGN
189	ENSG00000262209	4.05328324	18.7542855	-2.1762912	0.01180303	PCDHGB3	5	protein_coding	protocadherin gamma subfamily B, 3 [Source:HGNC Syn
190	ENSG00000277978	4.05328324	18.7542855	-2.1762912	0.01180303	AC010542.5	16	antisense	novel transcript, antisense to CKLF
191	ENSG00000269069	4.05328324	18.7542855	-2.1762912	0.01180303	AC007842.1	19	processed_pseud	protein associated with topoisomerase II homolog 1 (ye
192	ENSG00000142583	2.02720959	13.8186597	-2.6965904	0.01183243	SLC2A5	1	protein_coding	solute carrier family 2 member 5 [Source:HGNC Symbol;
193	ENSG00000251611	2.02720959	13.8186597	-2.6965904	0.01183243	FAM160A1-D	4	lincRNA	FAM160A1 divergent transcript [Source:HGNC Symbol;A
194	ENSG00000260417	2.02720959	13.8186597	-2.6965904	0.01183243	AC092127.1	16	lincRNA	novel transcript
195	ENSG00000279345	2.02720959	13.8186597	-2.6965904	0.01183243	Z98885.3	22	antisense	novel transcript, antisense to BRD1
196	ENSG00000158292	20.2619587	54.2907814	-1.4164852	0.0121478	GPR153	1	protein_coding	G protein-coupled receptor 153 [Source:HGNC Symbol;A
197	ENSG00000244932	20.2619587	54.2907814	-1.4164852	0.0121478	AL449212.1	3	unprocessed_pse	pseudogene similar to intraflagellar transport 122 hom
198	ENSG00000179583	20.2619587	54.2907814	-1.4164852	0.0121478	CIITA	16	protein_coding	class II major histocompatibility complex transactivator
199	ENSG00000176222	8.10544673	27.6384104	-1.7543928	0.0124954	ZNF404	19	protein_coding	zinc finger protein 404 [Source:HGNC Symbol;Acc:HGNC
200	ENSG00000198483	1.39E-17	7.89590589	-6.0040809	0.01273085	ANKRD35	1	protein_coding	ankyrin repeat domain 35 [Source:HGNC Symbol;Acc:HG

201	ENSG00000272750	1.39E-17	7.89590589	-6.0040809	0.01273085	AL592148.3	1	antisense	novel transcript, antisense to MIA3
202	ENSG00000172985	1.39E-17	7.89590589	-6.0040809	0.01273085	SH3RF3	2	protein_coding	SH3 domain containing ring finger 3 [Source:HGNC S
203	ENSG00000199121	1.39E-17	7.89590589	-6.0040809	0.01273085	MIR26B	2	miRNA	microRNA 26b [Source:HGNC Symbol;Acc:HGNC:316
204	ENSG00000078081	1.39E-17	7.89590589	-6.0040809	0.01273085	LAMP3	3	protein_coding	lysosomal associated membrane protein 3 [Source:H
205	ENSG00000205940	1.39E-17	7.89590589	-6.0040809	0.01273085	HSP90AB2P	4	transcribed_proc	heat shock protein 90 alpha family class B member
206	ENSG00000251526	1.39E-17	7.89590589	-6.0040809	0.01273085	LINC02435	4	lincRNA	long intergenic non-protein coding RNA 2435 [Sourc
207	ENSG00000274721	1.39E-17	7.89590589	-6.0040809	0.01273085	RF02039	5	misc_RNA	
208	ENSG00000229390	1.39E-17	7.89590589	-6.0040809	0.01273085	MICD	6	unprocessed_pse	MHC class I polypeptide-related sequence D (pseud
209	ENSG00000229452	1.39E-17	7.89590589	-6.0040809	0.01273085	AC005162.1	7	antisense	uncharacterized LOC107986703 [Source:NCBI gene;
210	ENSG00000235379	1.39E-17	7.89590589	-6.0040809	0.01273085	RPL7P31	7	processed_pseud	ribosomal protein L7 pseudogene 31 [Source:HGNC
211	ENSG00000259294	1.39E-17	7.89590589	-6.0040809	0.01273085	AC005096.1	7	antisense	novel transcript, antisense to CUX1
212	ENSG00000206685	1.39E-17	7.89590589	-6.0040809	0.01273085	RNU6-1189P	X	snRNA	RNA, U6 small nuclear 1189, pseudogene [Source:H
213	ENSG00000254274	1.39E-17	7.89590589	-6.0040809	0.01273085	TDGF1P5	8	processed_pseud	teratocarcinoma-derived growth factor 1 pseudogen
214	ENSG00000236745	1.39E-17	7.89590589	-6.0040809	0.01273085	YRDCP2	9	processed_pseud	yrdC N(6)-threonylcarbamoyltransferase domain cor
215	ENSG00000171671	1.39E-17	7.89590589	-6.0040809	0.01273085	SHANK2-AS3	11	antisense	SHANK2 antisense RNA 3 [Source:HGNC Symbol;Acc
216	ENSG00000213731	1.39E-17	7.89590589	-6.0040809	0.01273085	RAB5CP1	10	processed_pseud	RAB5C, member RAS oncogene family pseudogene 1
217	ENSG00000237280	1.39E-17	7.89590589	-6.0040809	0.01273085	AL136982.3	10	sense_intronic	novel transcript
218	ENSG00000277342	1.39E-17	7.89590589	-6.0040809	0.01273085	AC048344.4	12	sense_intronic	novel transcript, sense intronic to BICD1
219	ENSG00000251893	1.39E-17	7.89590589	-6.0040809	0.01273085	RF00156	12	snoRNA	
220	ENSG00000275880	1.39E-17	7.89590589	-6.0040809	0.01273085	AL139385.1	13	antisense	novel transcript, antisense to CARKD
221	ENSG00000275910	1.39E-17	7.89590589	-6.0040809	0.01273085	AC138932.5	16	antisense	novel transcript, antisense to PDXDC1
222	ENSG00000155714	1.39E-17	7.89590589	-6.0040809	0.01273085	PDZD9	16	protein_coding	PDZ domain containing 9 [Source:HGNC Symbol;Acc:H
223	ENSG00000277954	1.39E-17	7.89590589	-6.0040809	0.01273085	AC092376.2	16	antisense	novel transcript, antisense to WWOX
224	ENSG00000272636	1.39E-17	7.89590589	-6.0040809	0.01273085	DOC2B	17	protein_coding	double C2 domain beta [Source:HGNC Symbol;Acc:HGI
225	ENSG00000089558	1.39E-17	7.89590589	-6.0040809	0.01273085	KCNH4	17	protein_coding	potassium voltage-gated channel subfamily H membe
226	ENSG00000267432	1.39E-17	7.89590589	-6.0040809	0.01273085	DNAH17-AS1	17	processed_transc	DNAH17 antisense RNA 1 [Source:HGNC Symbol;Acc:H
227	ENSG00000267251	1.39E-17	7.89590589	-6.0040809	0.01273085	AC139100.1	18	antisense	novel transcript, antisense to PARD6G
228	ENSG00000228812	1.39E-17	7.89590589	-6.0040809	0.01273085	LAMA5-AS1	20	antisense	LAMA5 antisense RNA 1 [Source:HGNC Symbol;Acc:HG
229	ENSG00000279203	1.39E-17	7.89590589	-6.0040809	0.01273085	AC005785.2	19	TEC	TEC
230	ENSG00000274080	37.4836908	90.8143988	-1.2738987	0.01292629	AC005089.1	7	sense_intronic	novel transcript
231	ENSG00000243716	832.722574	1711.67327	-1.0393911	0.01317934	NPIPB5	16	protein_coding	nuclear pore complex interacting protein family memb
232	ENSG00000162975	5.06632294	20.7285356	-2.0065028	0.01323105	KCNF1	2	protein_coding	potassium voltage-gated channel modifier subfamily F
233	ENSG00000224775	5.06632294	20.7285356	-2.0065028	0.01323105	BRAFP1	X	unprocessed_pse	BRAF pseudogene 1 [Source:HGNC Symbol;Acc:HGNC:
234	ENSG00000255968	5.06632294	20.7285356	-2.0065028	0.01323105	AC024145.1	12	antisense	novel transcript to ITPR2
235	ENSG00000258317	5.06632294	20.7285356	-2.0065028	0.01323105	AC034102.6	12	antisense	novel transcript, antisense to ZC3H10 and ESYT1
236	ENSG00000242950	1.01417189	10.8572834	-3.2707058	0.01341209	ERVW-1	7	protein_coding	endogenous retrovirus group W member 1, envelope [
237	ENSG00000172476	1.01417189	10.8572834	-3.2707058	0.01341209	RAB40A	X	protein_coding	RAB40A, member RAS oncogene family [Source:HGNC
238	ENSG00000254207	1.01417189	10.8572834	-3.2707058	0.01341209	AC100797.1	8	sense_intronic	novel transcript
239	ENSG00000230724	1.01417189	10.8572834	-3.2707058	0.01341209	LINC01001	11	lincRNA	long intergenic non-protein coding RNA 1001 [Source:I
240	ENSG00000229990	1.01417189	10.8572834	-3.2707058	0.01341209	AC022400.2	10	antisense	novel transcript, antisense to CAMK2G

241	ENSG00000269938	1.01417189	10.8572834	-3.2707058	0.01341209	AC068790.2	12	sense_intronic	novel transcript, sense intronic to CCDC92
242	ENSG00000258648	1.01417189	10.8572834	-3.2707058	0.01341209	UBE2CP1	14	processed_pseud	ubiquitin conjugating enzyme E2 C pseudogene 1 [Sourc
243	ENSG00000232043	1.01417189	10.8572834	-3.2707058	0.01341209	AL133230.1	20	antisense	novel transcript, antisense to PTPN1
244	ENSG00000167642	1.01417189	10.8572834	-3.2707058	0.01341209	SPINT2	19	protein_coding	serine peptidase inhibitor, Kunitz type 2 [Source:HGNC S
245	ENSG00000064300	127.644531	274.419607	-1.1035148	0.01359801	NGFR	17	protein_coding	nerve growth factor receptor [Source:HGNC Symbol;Acc:
246	ENSG00000254681	721.287826	1471.80195	-1.0288123	0.01420726	PKD1P5	16	transcribed_unpr	polycystin 1, transient receptor potential channel intera
247	ENSG00000113205	3.04024516	15.7929101	-2.3308727	0.01448543	PCDHB3	5	protein_coding	protocadherin beta 3 [Source:HGNC Symbol;Acc:HGNC:8
248	ENSG00000269318	3.04024516	15.7929101	-2.3308727	0.01448543	AC007292.2	19	antisense	novel transcript, antisense to MPND
249	ENSG00000103657	265.418402	546.866043	-1.0425783	0.01491673	HERC1	15	protein_coding	HECT and RLD domain containing E3 ubiquitin protein li
250	ENSG00000170379	13.1706587	37.5096592	-1.501268	0.01493103	TCAF2	7	protein_coding	TRPM8 channel associated factor 2 [Source:HGNC Symb
251	ENSG00000080603	160.061913	334.634218	-1.063382	0.01518967	SRCAP	16	protein_coding	Snf2 related CREBBP activator protein [Source:HGNC Syr
252	ENSG00000205129	7.09240497	24.6770356	-1.7811914	0.01530539	C4orf47	4	protein_coding	chromosome 4 open reading frame 47 [Source:HGNC Sy
253	ENSG00000204710	7.09240497	24.6770356	-1.7811914	0.01530539	SPDYC	11	protein_coding	speedy/RINGO cell cycle regulator family member C [So
254	ENSG00000128011	7.09240497	24.6770356	-1.7811914	0.01530539	LRFN1	19	protein_coding	leucine rich repeat and fibronectin type III domain conta
255	ENSG00000185507	15.1967442	42.4452835	-1.4744067	0.01548729	IRF7	11	protein_coding	interferon regulatory factor 7 [Source:HGNC Symbol;Acc
256	ENSG00000075461	15.1967442	42.4452835	-1.4744067	0.01548729	CACNG4	17	protein_coding	calcium voltage-gated channel auxiliary subunit gamma
257	ENSG00000269901	16.209787	44.4195331	-1.4474332	0.01570992	AC010531.5	16	antisense	novel transcript, antisense to FBXO31
258	ENSG00000154237	41.5358633	96.7371475	-1.2172944	0.01571107	LRRK1	15	protein_coding	leucine rich repeat kinase 1 [Source:HGNC Symbol;Acc:H
259	ENSG00000244687	59.7706398	133.260764	-1.15512	0.01598344	UBE2V1	20	protein_coding	ubiquitin conjugating enzyme E2 V1 [Source:HGNC Symb
260	ENSG00000229167	18.2358728	48.3680324	-1.4012616	0.01607015	AC114488.1	1	antisense	novel transcript
261	ENSG00000159433	270.483618	553.775917	-1.0334288	0.01622135	STARD9	15	protein_coding	StAR related lipid transfer domain containing 9 [Source
262	ENSG00000072840	220.844502	454.076315	-1.0394952	0.01631141	EVC	4	protein_coding	EvC ciliary complex subunit 1 [Source:HGNC Symbol;Ac
263	ENSG00000079308	1188.30073	2380.94386	-1.0025596	0.01631189	TNS1	2	protein_coding	tensin 1 [Source:HGNC Symbol;Acc:HGNC:11973]
264	ENSG00000275747	4.05328324	17.7671604	-2.0988192	0.01649288	IGHV3-79	14	IG_V_pseudogen	immunoglobulin heavy variable 3-79 (pseudogene) [Sou
265	ENSG00000228218	4.05328324	17.7671604	-2.0988192	0.01649288	ATF4P3	17	processed_pseud	activating transcription factor 4 pseudogene 3 [Source:
266	ENSG00000142920	26.3402168	64.1620294	-1.2805145	0.01682585	AZIN2	1	protein_coding	antizyme inhibitor 2 [Source:HGNC Symbol;Acc:HGNC:2
267	ENSG00000260428	28.3663029	68.1105286	-1.2600801	0.01691656	SCX	8	protein_coding	scleraxis bHLH transcription factor [Source:HGNC Symb
268	ENSG00000180747	124.605402	260.59986	-1.0637337	0.01720046	SMG1P3	16	transcribed_unpr	SMG1 pseudogene 3 [Source:HGNC Symbol;Acc:HGNC:4
269	ENSG00000257596	12.157616	34.5482846	-1.4973814	0.01786044	AC078778.1	12	antisense	novel transcript, antisense to CBX5
270	ENSG00000235908	2.02720959	12.8315343	-2.5906695	0.01786222	RHOA-IT1	3	sense_intronic	RHOA intronic transcript 1 [Source:HGNC Symbol;Acc:H
271	ENSG00000277968	2.02720959	12.8315343	-2.5906695	0.01786222	RF02038	5	misc_RNA	
272	ENSG00000231701	2.02720959	12.8315343	-2.5906695	0.01786222	BMS1P13	9	unprocessed_pse	BMS1, ribosome biogenesis factor pseudogene 13 [Sou
273	ENSG00000262152	2.02720959	12.8315343	-2.5906695	0.01786222	LINC00514	16	processed_transc	long intergenic non-protein coding RNA 514 [Source:HG
274	ENSG00000126861	2.02720959	12.8315343	-2.5906695	0.01786222	OMG	17	protein_coding	oligodendrocyte myelin glycoprotein [Source:HGNC Syn
275	ENSG00000278829	2.02720959	12.8315343	-2.5906695	0.01786222	AC099811.5	17	sense_intronic	novel transcript, sense intronic to STAT5B
276	ENSG00000265678	2.02720959	12.8315343	-2.5906695	0.01786222	AC129510.1	17	antisense	novel transcript, antisense CCDC57
277	ENSG00000272491	5.06632294	19.7414106	-1.9365457	0.01800317	AL109659.2	1	antisense	novel transcript, antisense to SLC5A9
278	ENSG00000137496	88.1358481	185.578377	-1.0731809	0.01848975	IL18BP	11	protein_coding	interleukin 18 binding protein [Source:HGNC Symbol;Ac
279	ENSG00000185615	15.1967442	40.4710338	-1.4059006	0.01850271	PDIA2	16	protein_coding	protein disulfide isomerase family A member 2 [Source
280	ENSG00000260029	15.1967442	40.4710338	-1.4059006	0.01850271	AC007608.2	16	antisense	novel transcript, antisense to NKD1

281	ENSG00000183153	16.209787	43.4324083	-1.4151037	0.01863969	GJD3	17	protein_coding	gap junction protein delta 3 [Source:HGNC Symbol;A
282	ENSG00000156966	19.2489157	49.3551573	-1.3528481	0.01890245	B3GNT7	2	protein_coding	UDP-GlcNAc:betaGal beta-1,3-N-acetylglucosaminyl
283	ENSG00000112276	25.3271738	61.200655	-1.2687897	0.01904724	BVES	6	protein_coding	blood vessel epicardial substance [Source:HGNC Syr
284	ENSG00000228060	6.07936364	21.7156606	-1.8159916	0.01913737	AL365277.1	1	antisense	uncharacterized LOC101929516 [Source:NCBI gene;/
285	ENSG00000260005	6.07936364	21.7156606	-1.8159916	0.01913737	AC027601.1	17	antisense	uncharacterized LOC105371925 [Source:NCBI gene;/
286	ENSG00000188167	100.292366	208.282247	-1.0534199	0.01980039	TMPPE	3	protein_coding	transmembrane protein with metallophosphoesteras
287	ENSG00000253372	7.09240497	23.6899106	-1.7226006	0.01998788	AC016405.1	8	sense_intronic	novel transcript
288	ENSG00000047662	3.04024516	14.8057849	-2.2385182	0.02095211	FAM184B	4	protein_coding	family with sequence similarity 184 member B [Sour
289	ENSG00000262558	3.04024516	14.8057849	-2.2385182	0.02095211	AC129507.3	17	antisense	novel transcript, antisense to RPH3AL
290	ENSG00000229222	3.04024516	14.8057849	-2.2385182	0.02095211	KRT18P4	20	processed_pseud	keratin 18 pseudogene 4 [Source:HGNC Symbol;Acc:
291	ENSG00000228817	3.04024516	14.8057849	-2.2385182	0.02095211	BACH1-IT2	21	lincRNA	BACH1 intronic transcript 2 [Source:HGNC Symbol;A
292	ENSG00000275131	32.4184753	74.0332774	-1.1883092	0.02107841	AC241952.1	1	transcribed_unpr	phosphodiesterase 4D interacting protein-like [Sour
293	ENSG00000146197	32.4184753	74.0332774	-1.1883092	0.02107841	SCUBE3	6	protein_coding	signal peptide, CUB domain and EGF like domain co
294	ENSG00000153531	9.11848879	27.6384104	-1.5869045	0.02109633	ADPRHL1	13	protein_coding	ADP-ribosylhydrolase like 1 [Source:HGNC Symbol;A
295	ENSG00000279801	9.11848879	27.6384104	-1.5869045	0.02109633	AC111170.3	17	TEC	TEC
296	ENSG00000285701	1.01417189	9.87015775	-3.1348432	0.02156655	AL357140.5	1	antisense	novel transcript, antisense to CTNNBIP1
297	ENSG00000275557	1.01417189	9.87015775	-3.1348432	0.02156655	AC242842.1	1	processed_transc	novel transcript
298	ENSG00000214837	1.01417189	9.87015775	-3.1348432	0.02156655	LINC01347	1	transcribed_unpr	long intergenic non-protein coding RNA 1347 [Sourc
299	ENSG00000224618	1.01417189	9.87015775	-3.1348432	0.02156655	AC096921.1	3	processed_pseud	ectodermal-neural cortex (ENC1) pseudogene
300	ENSG00000280417	1.01417189	9.87015775	-3.1348432	0.02156655	AC096887.2	3	TEC	TEC
301	ENSG00000248774	1.01417189	9.87015775	-3.1348432	0.02156655	AC097534.1	4	antisense	novel transcript
302	ENSG00000204758	1.01417189	9.87015775	-3.1348432	0.02156655	AC008429.1	5	antisense	uncharacterized LOC100268168 [Source:NCBI gene;Ac
303	ENSG00000199332	1.01417189	9.87015775	-3.1348432	0.02156655	RF00019	6	misc_RNA	
304	ENSG00000228653	1.01417189	9.87015775	-3.1348432	0.02156655	HNRNPCP7	7	processed_pseud	heterogeneous nuclear ribonucleoprotein C pseudogen
305	ENSG00000242798	1.01417189	9.87015775	-3.1348432	0.02156655	AC073842.2	7	antisense	novel transcript
306	ENSG00000229413	1.01417189	9.87015775	-3.1348432	0.02156655	AC018638.1	7	processed_pseud	novel pseudogene
307	ENSG00000061337	1.01417189	9.87015775	-3.1348432	0.02156655	LZTS1	8	protein_coding	leucine zipper tumor suppressor 1 [Source:HGNC Symb
308	ENSG00000250569	1.01417189	9.87015775	-3.1348432	0.02156655	NTAN1P2	8	processed_pseud	N-terminal asparagine amidase pseudogene 2 [Source:
309	ENSG00000278988	1.01417189	9.87015775	-3.1348432	0.02156655	AL356490.1	9	TEC	tec
310	ENSG00000228828	1.01417189	9.87015775	-3.1348432	0.02156655	TLK2P2	10	processed_pseud	tousled like kinase 2 pseudogene 2 [Source:HGNC Sym
311	ENSG00000232470	1.01417189	9.87015775	-3.1348432	0.02156655	AL136368.1	10	lincRNA	novel transcript
312	ENSG00000258048	1.01417189	9.87015775	-3.1348432	0.02156655	AC073569.2	12	lincRNA	novel transcript
313	ENSG00000169508	1.01417189	9.87015775	-3.1348432	0.02156655	GPR183	13	protein_coding	G protein-coupled receptor 183 [Source:HGNC Symbol;/
314	ENSG00000273618	1.01417189	9.87015775	-3.1348432	0.02156655	AL135999.2	14	antisense	TEC
315	ENSG00000237356	1.01417189	9.87015775	-3.1348432	0.02156655	AL365295.1	14	processed_transc	novel transcript
316	ENSG00000226132	1.01417189	9.87015775	-3.1348432	0.02156655	RPS3AP46	14	processed_pseud	ribosomal protein S3a pseudogene 46 [Source:HGNC Sy
317	ENSG00000169918	1.01417189	9.87015775	-3.1348432	0.02156655	OTUD7A	15	protein_coding	OTU deubiquitinase 7A [Source:HGNC Symbol;Acc:HGN
318	ENSG00000276384	1.01417189	9.87015775	-3.1348432	0.02156655	AC016876.3	17	antisense	novel transcript, antisense to TNFSF12
319	ENSG00000279281	1.01417189	9.87015775	-3.1348432	0.02156655	AC015883.1	17	TEC	TEC
320	ENSG00000280486	1.01417189	9.87015775	-3.1348432	0.02156655	AC005329.3	19	antisense	novel transcript, antisense to NDUFS7

321	ENSG00000236890	1.01417189	9.87015775	-3.1348432	0.02156655	AC016027.5	22	processed_pseud	ribosomal protein L18a (RPL18A) pseu
322	ENSG00000227192	25.3271738	60.2135302	-1.2453789	0.02163149	AL023581.2	6	lincRNA	novel transcript
323	ENSG00000203865	11.1445735	31.58691	-1.4927826	0.02169529	ATP1A1-AS1	1	processed_transc	ATP1A1 antisense RNA 1 [Source:HGN
324	ENSG00000176920	11.1445735	31.58691	-1.4927826	0.02169529	FUT2	19	protein_coding	fucosyltransferase 2 [Source:HGNC Sy
325	ENSG00000228624	12.157616	33.5611598	-1.4557142	0.02187215	HDAC2-AS2	6	antisense	HDAC2 and HS3ST5 antisense RNA 2 [
326	ENSG00000277496	12.157616	33.5611598	-1.4557142	0.02187215	AL357033.4	20	antisense	novel transcript, antisense to SLCO4A1
327	ENSG00000226363	21.2750017	52.3165317	-1.2932029	0.02191967	HAGLROS	2	lincRNA	HAGLR opposite strand lncRNA [Source
328	ENSG00000215156	13.1706587	35.5354095	-1.4235316	0.02199107	AC138409.1	5	processed_pseud	integral membrane glycoprotein-like [S
329	ENSG00000187800	15.1967442	39.4839089	-1.3703877	0.02210299	PEAR1	1	protein_coding	platelet endothelial aggregation recep
330	ENSG00000206651	1.39E-17	6.90877955	-5.8146434	0.02267682	RF00019	1	misc_RNA	
331	ENSG00000231084	1.39E-17	6.90877955	-5.8146434	0.02267682	RPL22P24	1	processed_pseud	ribosomal protein L22 pseudogene 24
332	ENSG00000162849	1.39E-17	6.90877955	-5.8146434	0.02267682	KIF26B	1	protein_coding	kinesin family member 26B [Source:HG
333	ENSG00000115353	1.39E-17	6.90877955	-5.8146434	0.02267682	TACR1	2	protein_coding	tachykinin receptor 1 [Source:HGNC Sy
334	ENSG00000114948	1.39E-17	6.90877955	-5.8146434	0.02267682	ADAM23	2	protein_coding	ADAM metallopeptidase domain 23 [S
335	ENSG00000225493	1.39E-17	6.90877955	-5.8146434	0.02267682	LINC01107	2	lincRNA	long intergenic non-protein coding RN
336	ENSG00000172020	1.39E-17	6.90877955	-5.8146434	0.02267682	GAP43	3	protein_coding	growth associated protein 43 [Source:I
337	ENSG00000249047	1.39E-17	6.90877955	-5.8146434	0.02267682	COX6B1P5	4	processed_pseud	cytochrome c oxidase subunit 6B1 pse
338	ENSG00000279481	1.39E-17	6.90877955	-5.8146434	0.02267682	AC104791.2	4	TEC	TEC
339	ENSG00000272123	1.39E-17	6.90877955	-5.8146434	0.02267682	AC008966.2	5	antisense	novel transcript, antisense to ITGA2
340	ENSG00000253333	1.39E-17	6.90877955	-5.8146434	0.02267682	AC146944.3	5	unprocessed_pse	glucuronidase, beta (GUSB) pseudoger
341	ENSG00000120329	1.39E-17	6.90877955	-5.8146434	0.02267682	SLC25A2	5	protein_coding	solute carrier family 25 member 2 [Sourc
342	ENSG00000236714	1.39E-17	6.90877955	-5.8146434	0.02267682	LINC01844	5	lincRNA	long intergenic non-protein coding RNA
343	ENSG00000277039	1.39E-17	6.90877955	-5.8146434	0.02267682	RF00017	5	misc_RNA	
344	ENSG00000124493	1.39E-17	6.90877955	-5.8146434	0.02267682	GRM4	6	protein_coding	glutamate metabotropic receptor 4 [Sour
345	ENSG00000279659	1.39E-17	6.90877955	-5.8146434	0.02267682	AL451064.2	6	TEC	TEC
346	ENSG00000226310	1.39E-17	6.90877955	-5.8146434	0.02267682	AL022157.1	X	antisense	novel transcript
347	ENSG00000172733	1.39E-17	6.90877955	-5.8146434	0.02267682	PURG	8	protein_coding	purine rich element binding protein G [S
348	ENSG00000254388	1.39E-17	6.90877955	-5.8146434	0.02267682	DUTP2	8	processed_pseud	deoxyuridine triphosphatase pseudogen
349	ENSG00000279786	1.39E-17	6.90877955	-5.8146434	0.02267682	AC105235.1	8	TEC	TEC
350	ENSG00000254973	1.39E-17	6.90877955	-5.8146434	0.02267682	AC105219.3	8	lincRNA	novel transcript
351	ENSG00000183784	1.39E-17	6.90877955	-5.8146434	0.02267682	C9orf66	9	protein_coding	chromosome 9 open reading frame 66 [S
352	ENSG00000225408	1.39E-17	6.90877955	-5.8146434	0.02267682	AL136980.1	9	antisense	novel transcript
353	ENSG00000231697	1.39E-17	6.90877955	-5.8146434	0.02267682	NANOGP5	9	transcribed_proc	Nanog homeobox pseudogene 5 [Source
354	ENSG00000213221	1.39E-17	6.90877955	-5.8146434	0.02267682	DNLZ	9	protein_coding	DNL-type zinc finger [Source:HGNC Symb
355	ENSG00000236394	1.39E-17	6.90877955	-5.8146434	0.02267682	AL807752.4	9	antisense	uncharacterized LOC105376327 [Source:
356	ENSG00000225140	1.39E-17	6.90877955	-5.8146434	0.02267682	AL358216.1	10	antisense	novel transcript
357	ENSG00000185933	1.39E-17	6.90877955	-5.8146434	0.02267682	CALHM1	10	protein_coding	calcium homeostasis modulator 1 [Sourc
358	ENSG00000188916	1.39E-17	6.90877955	-5.8146434	0.02267682	INSYN2	10	protein_coding	inhibitory synaptic factor 2A [Source:HG
359	ENSG00000174527	1.39E-17	6.90877955	-5.8146434	0.02267682	MYO1H	12	protein_coding	myosin IH [Source:HGNC Symbol;Acc:HG
360	ENSG00000273784	1.39E-17	6.90877955	-5.8146434	0.02267682	AL137058.2	13	processed_transc	novel transcript

361	ENSG00000254656	1.39E-17	6.90877955	-5.8146434	0.02267682	RTL1	14	protein_coding	retrotransposon Gag like 1 [Source:HGNC
362	ENSG00000259654	1.39E-17	6.90877955	-5.8146434	0.02267682	AC115102.1	15	antisense	novel transcript, antisense to SEC11A
363	ENSG00000225193	1.39E-17	6.90877955	-5.8146434	0.02267682	RPS12P26	15	transcribed_proc	ribosomal protein S12 pseudogene 26 [Sc
364	ENSG00000127588	1.39E-17	6.90877955	-5.8146434	0.02267682	GNG13	16	protein_coding	G protein subunit gamma 13 [Source:HGN
365	ENSG00000279901	1.39E-17	6.90877955	-5.8146434	0.02267682	AC092117.2	16	TEC	tec
366	ENSG00000154102	1.39E-17	6.90877955	-5.8146434	0.02267682	C16orf74	16	protein_coding	chromosome 16 open reading frame 74 [
367	ENSG00000267048	1.39E-17	6.90877955	-5.8146434	0.02267682	AC092143.3	16	processed_transc	novel transcript (MC1R-TUBB3 readthrou
368	ENSG00000181885	1.39E-17	6.90877955	-5.8146434	0.02267682	CLDN7	17	protein_coding	claudin 7 [Source:HGNC Symbol;Acc:HGN
369	ENSG00000262298	1.39E-17	6.90877955	-5.8146434	0.02267682	AC004584.1	17	antisense	novel transcript, antisense to COIL
370	ENSG00000267529	1.39E-17	6.90877955	-5.8146434	0.02267682	AP005131.4	18	processed_transc	novel transcript, antisense C18orf1
371	ENSG00000278075	1.39E-17	6.90877955	-5.8146434	0.02267682	AC114341.1	18	lincRNA	novel transcript
372	ENSG00000104951	1.39E-17	6.90877955	-5.8146434	0.02267682	IL4I1	19	protein_coding	interleukin 4 induced 1 [Source:HGNC Syr
373	ENSG00000273032	1.39E-17	6.90877955	-5.8146434	0.02267682	DGCR9	22	processed_transc	DiGeorge syndrome critical region gene 9
374	ENSG00000073146	1.39E-17	6.90877955	-5.8146434	0.02267682	MOV10L1	22	protein_coding	Mov10 like RISC complex RNA helicase 1
375	ENSG00000260948	4.05328324	16.7800353	-2.0169497	0.02307085	AL390195.2	1	sense_overlappir	novel transcript, sense overlapping WDR
376	ENSG00000207280	4.05328324	16.7800353	-2.0169497	0.02307085	SNORD20	2	snoRNA	small nucleolar RNA, C/D box 20 [Source:
377	ENSG00000201207	4.05328324	16.7800353	-2.0169497	0.02307085	RF00019	6	misc_RNA	
378	ENSG00000184937	4.05328324	16.7800353	-2.0169497	0.02307085	WT1	11	protein_coding	Wilms tumor 1 [Source:HGNC Symbol;Acc
379	ENSG00000105695	4.05328324	16.7800353	-2.0169497	0.02307085	MAG	19	protein_coding	myelin associated glycoprotein [Source:H
380	ENSG00000197283	93.2010639	189.526876	-1.0230269	0.0239684	SYNGAP1	6	protein_coding	synaptic Ras GTPase activating protein 1
381	ENSG00000279602	5.06632294	18.7542855	-1.8630228	0.0245106	AC109326.1	17	TEC	TEC
382	ENSG00000164142	60.7836829	128.32514	-1.0765268	0.0249924	FAM160A1	4	protein_coding	family with sequence similarity 160 member
383	ENSG00000246575	46.6010789	100.685647	-1.1093975	0.02542063	AC093162.2	2	unprocessed_pse	retinol saturase (all-trans-retinol 13,14-reduc
384	ENSG00000227959	6.07936364	20.7285356	-1.7492705	0.0254753	AL451042.2	1	antisense	novel transcript
385	ENSG00000207554	19.2489157	47.3809076	-1.2941062	0.02567534	MIR647	20	miRNA	microRNA 647 [Source:HGNC Symbol;Acc:HGI
386	ENSG00000231240	17.2228299	43.4324083	-1.3282904	0.02605029	KLF2P1	2	processed_pseuc	Kruppel like factor 2 pseudogene 1 [Source:H
387	ENSG00000205041	17.2228299	43.4324083	-1.3282904	0.02605029	AC118344.1	19	sense_intronic	novel transcript, sense intronic to AKT2
388	ENSG00000145020	7.09240497	22.7027856	-1.6615292	0.02610533	AMT	3	protein_coding	aminomethyltransferase [Source:HGNC Symb
389	ENSG00000283128	7.09240497	22.7027856	-1.6615292	0.02610533	AC009403.2	7	processed_transc	novel transcript
390	ENSG00000279605	7.09240497	22.7027856	-1.6615292	0.02610533	AC067930.6	8	TEC	TEC
391	ENSG00000071246	7.09240497	22.7027856	-1.6615292	0.02610533	VASH1	14	protein_coding	vasohibin 1 [Source:HGNC Symbol;Acc:HGNC:
392	ENSG00000197744	16.209787	40.4710338	-1.313527	0.02622973	PTMAP2	5	processed_pseuc	prothymosin alpha pseudogene 2 [Source:HG
393	ENSG00000179776	16.209787	40.4710338	-1.313527	0.02622973	CDH5	16	protein_coding	cadherin 5 [Source:HGNC Symbol;Acc:HGNC:1
394	ENSG00000198342	14.1837014	36.5225344	-1.3569773	0.02655207	ZNF442	19	protein_coding	zinc finger protein 442 [Source:HGNC Symbol;
395	ENSG00000276603	9.11848879	26.6512854	-1.5346776	0.02672002	AL109614.1	20	sense_intronic	novel transcript, sense intronic to BLCAP
396	ENSG00000114670	12.157616	32.5740349	-1.4128077	0.02678003	NEK11	3	protein_coding	NIMA related kinase 11 [Source:HGNC Symbo
397	ENSG00000163596	29.379346	66.136279	-1.1673159	0.0267866	ICA1L	2	protein_coding	islet cell autoantigen 1 like [Source:HGNC Syr
398	ENSG00000241404	10.1315311	28.6255353	-1.4872559	0.02682007	EGFL8	6	protein_coding	EGF like domain multiple 8 [Source:HGNC Syr
399	ENSG00000151320	10.1315311	28.6255353	-1.4872559	0.02682007	AKAP6	14	protein_coding	A-kinase anchoring protein 6 [Source:HGNC S
400	ENSG00000261654	2.02720959	11.8444089	-2.4763515	0.02704271	AL360270.2	1	sense_overlappir	novel transcript, sense overlapping C1orf103

401	ENSG00000249084	2.02720959	11.8444089	-2.4763515	0.02704271	AC027627.1	4	antisense	novel transcript
402	ENSG00000227351	2.02720959	11.8444089	-2.4763515	0.02704271	NANOGP6	10	processed_pseud	Nanog homeobox pseudogene 6 [Source:HGNC Symbol
403	ENSG00000258735	2.02720959	11.8444089	-2.4763515	0.02704271	LINC00637	14	lincRNA	long intergenic non-protein coding RNA 637 [Source:H(
404	ENSG00000265845	2.02720959	11.8444089	-2.4763515	0.02704271	AC024267.4	17	antisense	uncharacterized LOC101927018 [Source:NCBI gene;Ac
405	ENSG00000213228	2.02720959	11.8444089	-2.4763515	0.02704271	RPL12P38	17	transcribed_proc	ribosomal protein L12 pseudogene 38 [Source:HGNC S
406	ENSG00000265912	2.02720959	11.8444089	-2.4763515	0.02704271	AC037487.1	17	lincRNA	novel transcript
407	ENSG00000267934	2.02720959	11.8444089	-2.4763515	0.02704271	AC010300.1	19	antisense	novel transcript, antisense to ZNF91
408	ENSG00000187642	49.6402084	104.634146	-1.0739135	0.02894954	PERM1	1	protein_coding	PPARGC1 and ESRR induced regulator, muscle 1 [Sourc
409	ENSG00000273899	29.379346	65.1491542	-1.145662	0.03002198	NOL12	22	protein_coding	nucleolar protein 12 [Source:HGNC Symbol;Acc:HGNC:
410	ENSG00000258366	68.8880282	140.170638	-1.0235636	0.03015728	RTEL1	20	protein_coding	regulator of telomere elongation helicase 1 [Source:H(
411	ENSG00000269068	3.04024516	13.8186597	-2.1398449	0.03034815	AC009955.4	2	antisense	novel transcript, antisense to OBSL1
412	ENSG00000260651	3.04024516	13.8186597	-2.1398449	0.03034815	AF213884.3	4	antisense	novel transcript, antisense NFKB1
413	ENSG00000260996	3.04024516	13.8186597	-2.1398449	0.03034815	BX255925.1	9	sense_overlappir	novel transcript, overlapping NRARP
414	ENSG00000226627	3.04024516	13.8186597	-2.1398449	0.03034815	SHANK2-AS1	11	antisense	SHANK2 antisense RNA 1 [Source:HGNC Symbol;Acc:H(
415	ENSG00000227374	3.04024516	13.8186597	-2.1398449	0.03034815	AL157832.1	10	lincRNA	novel transcript
416	ENSG00000123009	3.04024516	13.8186597	-2.1398449	0.03034815	NME2P1	12	processed_pseud	NME/NM23 nucleoside diphosphate kinase 2 pseudog
417	ENSG00000242085	3.04024516	13.8186597	-2.1398449	0.03034815	RPS20P33	14	processed_pseud	ribosomal protein S20 pseudogene 33 [Source:HGNC S
418	ENSG00000257950	3.04024516	13.8186597	-2.1398449	0.03034815	P2RX5-TAX1E	17	protein_coding	P2RX5-TAX1BP3 readthrough (NMD candidate) [Source
419	ENSG00000167874	3.04024516	13.8186597	-2.1398449	0.03034815	TMEM88	17	protein_coding	transmembrane protein 88 [Source:HGNC Symbol;Acc:I
420	ENSG00000263006	3.04024516	13.8186597	-2.1398449	0.03034815	ROCK1P1	18	transcribed_unpr	Rho associated coiled-coil containing protein kinase 1
421	ENSG00000235065	3.04024516	13.8186597	-2.1398449	0.03034815	RPL24P2	20	processed_pseud	ribosomal protein L24 pseudogene 2 [Source:HGNC Sy
422	ENSG00000285979	63.8228124	130.29939	-1.0282835	0.03138531	AC009090.6	16	antisense	novel transcript, antisense to FAM192A
423	ENSG00000086730	15.1967442	37.5096592	-1.2966267	0.0315204	LAT2	7	protein_coding	linker for activation of T cells family member 2 [Sourc
424	ENSG00000050555	40.5228202	86.8658997	-1.0977434	0.03169889	LAMC3	9	protein_coding	laminin subunit gamma 3 [Source:HGNC Symbol;Acc:H
425	ENSG00000278467	14.1837014	35.5354095	-1.3175856	0.03194235	AC138393.3	1	lincRNA	novel transcript
426	ENSG00000279162	4.05328324	15.7929101	-1.9301534	0.03229224	AC141586.5	16	TEC	tec
427	ENSG00000167617	22.2880447	51.3294069	-1.1990572	0.03312109	CDC42EP5	19	protein_coding	CDC42 effector protein 5 [Source:HGNC Symbol;Acc:H
428	ENSG00000205913	11.1445735	29.6126602	-1.4000522	0.03317368	SRRM2-AS1	16	antisense	SRRM2 antisense RNA 1 [Source:HGNC Symbol;Acc:H(
429	ENSG00000254369	5.06632294	17.7671604	-1.7855507	0.03337634	HOXA-AS3	7	antisense	HOXA cluster antisense RNA 3 [Source:HGNC Symbol;.
430	ENSG00000168916	10.1315311	27.6384104	-1.4368538	0.03353309	ZNF608	5	protein_coding	zinc finger protein 608 [Source:HGNC Symbol;Acc:HGN
431	ENSG00000205414	29.379346	64.1620294	-1.1236782	0.03364177	AC007608.1	16	antisense	novel transcript, antisense to NKD1
432	ENSG00000279261	9.11848879	25.6641605	-1.4804888	0.03383233	AL360294.1	1	lincRNA	uncharacterized LOC100130249 [Source:NCBI gene;Ac
433	ENSG00000274317	9.11848879	25.6641605	-1.4804888	0.03383233	LINC02334	13	lincRNA	long intergenic non-protein coding RNA 2334 [Source:
434	ENSG00000237991	6.07936364	19.7414106	-1.6793134	0.03391022	RPL35P1	1	processed_pseud	ribosomal protein L35 pseudogene 1 [Source:HGNC Sy
435	ENSG00000216316	6.07936364	19.7414106	-1.6793134	0.03391022	AL022722.1	6	processed_pseud	60S Ribosomal Protein L13A (RPL13) pseudogene
436	ENSG00000180846	6.07936364	19.7414106	-1.6793134	0.03391022	CSNK1G2-AS1	19	antisense	CSNK1G2 antisense RNA 1 [Source:HGNC Symbol;Acc:
437	ENSG00000197536	8.10544673	23.6899106	-1.5330824	0.03403535	C5orf56	5	processed_transc	chromosome 5 open reading frame 56 [Source:HGNC !
438	ENSG00000214185	8.10544673	23.6899106	-1.5330824	0.03403535	XPOTP1	20	processed_pseud	exportin for tRNA pseudogene 1 [Source:HGNC Symbo
439	ENSG00000126500	7.09240497	21.7156606	-1.5977579	0.03408838	FLRT1	11	protein_coding	fibronectin leucine rich transmembrane protein 1 [Sou
440	ENSG00000138316	7.09240497	21.7156606	-1.5977579	0.03408838	ADAMTS14	10	protein_coding	ADAM metallopeptidase with thrombospondin type 1

441	ENSG00000258730	7.09240497	21.7156606	-1.5977579	0.03408838	ITPK1-AS1	14	antisense	ITPK1 antisense RNA 1 [Source:HGNC Sym
442	ENSG00000197670	28.3663029	62.1877798	-1.1290872	0.03416525	AL157838.1	20	antisense	uncharacterized LOC101927770 [Source:N
443	ENSG00000232545	35.4576046	75.0204022	-1.078578	0.03423895	AC253536.3	22	sense_intronic	novel transcript
444	ENSG00000237200	1.01417189	8.88303193	-2.984843	0.0348666	ZBTB40-IT1	1	sense_intronic	ZBTB40 intronic transcript 1 [Source:HGNC
445	ENSG00000226876	1.01417189	8.88303193	-2.984843	0.0348666	AC092801.1	1	antisense	novel transcript
446	ENSG00000285600	1.01417189	8.88303193	-2.984843	0.0348666	AC023593.1	3	antisense	
447	ENSG00000138646	1.01417189	8.88303193	-2.984843	0.0348666	HERC5	4	protein_coding	HECT and RLD domain containing E3 ubiqu
448	ENSG00000250138	1.01417189	8.88303193	-2.984843	0.0348666	AC139495.3	5	unprocessed_pse	POM121 membrane glycoprotein-like 1 (P(
449	ENSG00000253203	1.01417189	8.88303193	-2.984843	0.0348666	GUSBP3	5	transcribed_unpr	glucuronidase, beta pseudogene 3 [Source
450	ENSG00000129595	1.01417189	8.88303193	-2.984843	0.0348666	EPB41L4A	5	protein_coding	erythrocyte membrane protein band 4.1 lik
451	ENSG00000146006	1.01417189	8.88303193	-2.984843	0.0348666	LRRTM2	5	protein_coding	leucine rich repeat transmembrane neuror
452	ENSG00000247925	1.01417189	8.88303193	-2.984843	0.0348666	AL139807.1	6	antisense	novel transcript
453	ENSG00000274594	1.01417189	8.88303193	-2.984843	0.0348666	AL391669.1	6	processed_pseud	transmembrane protein 80 (TMEM80) pse
454	ENSG00000128596	1.01417189	8.88303193	-2.984843	0.0348666	CCDC136	7	protein_coding	coiled-coil domain containing 136 [Source:
455	ENSG00000246477	1.01417189	8.88303193	-2.984843	0.0348666	AF131216.1	8	antisense	novel transcript, antisense to MTMR9
456	ENSG00000227852	1.01417189	8.88303193	-2.984843	0.0348666	RPS29P17	9	processed_pseud	ribosomal protein S29 pseudogene 17 [Sou
457	ENSG00000173269	1.01417189	8.88303193	-2.984843	0.0348666	MMRN2	10	protein_coding	multimerin 2 [Source:HGNC Symbol;Acc:H(
458	ENSG00000273108	1.01417189	8.88303193	-2.984843	0.0348666	AL121929.2	10	antisense	novel transcript, antisense to SH3PXD2A
459	ENSG00000258302	1.01417189	8.88303193	-2.984843	0.0348666	AC025034.1	12	antisense	novel transcript, antisense to ATP2B1
460	ENSG00000277840	1.01417189	8.88303193	-2.984843	0.0348666	AC026368.1	12	sense_intronic	novel transcript, sense intronic to FBXW8
461	ENSG00000225083	1.01417189	8.88303193	-2.984843	0.0348666	GRTP1-AS1	13	antisense	GRTP1 antisense RNA 1 [Source:HGNC Sy
462	ENSG00000275454	1.01417189	8.88303193	-2.984843	0.0348666	AC105020.6	15	antisense	novel transcript, antisense to IMP3
463	ENSG00000260403	1.01417189	8.88303193	-2.984843	0.0348666	AC120498.3	16	antisense	novel transcript, antisense to CACNA1H
464	ENSG00000260979	1.01417189	8.88303193	-2.984843	0.0348666	AC022167.3	16	lincRNA	novel transcript
465	ENSG00000275441	1.01417189	8.88303193	-2.984843	0.0348666	AC020765.2	16	antisense	novel transcript, antisense to CCDC101
466	ENSG00000159618	1.01417189	8.88303193	-2.984843	0.0348666	ADGRG5	16	protein_coding	adhesion G protein-coupled receptor G5
467	ENSG00000266126	1.01417189	8.88303193	-2.984843	0.0348666	AC005730.3	17	sense_intronic	novel transcript
468	ENSG00000274776	1.01417189	8.88303193	-2.984843	0.0348666	AC090241.3	18	lincRNA	novel transcript
469	ENSG00000267006	1.01417189	8.88303193	-2.984843	0.0348666	AC008507.2	19	lincRNA	novel transcript
470	ENSG00000268756	1.01417189	8.88303193	-2.984843	0.0348666	AC104534.1	19	antisense	novel transcript, antisense to ECH1
471	ENSG00000219200	26.3402168	58.2392806	-1.1410749	0.03530381	RNASEK	17	protein_coding	ribonuclease K [Source:HGNC Symbol;Acc
472	ENSG00000204947	15.1967442	36.5225344	-1.258282	0.03762082	ZNF425	7	protein_coding	zinc finger protein 425 [Source:HGNC Syn
473	ENSG00000183562	15.1967442	36.5225344	-1.258282	0.03762082	AC131971.1	11	antisense	novel transcript, antisense to NAP1L4
474	ENSG00000143570	14.1837014	34.5482846	-1.277088	0.03841068	SLC39A1	1	protein_coding	solute carrier family 39 member 1 [Sourc
475	ENSG00000118263	34.4445615	72.0590278	-1.0622448	0.03842835	KLF7	2	protein_coding	Kruppel like factor 7 [Source:HGNC Symb
476	ENSG00000223813	21.2750017	48.3680324	-1.180273	0.03881785	AC007255.1	7	antisense	novel transcript, antisense to CHN2
477	ENSG00000187889	12.157616	30.5997851	-1.3229656	0.04010141	FYB2	1	protein_coding	FYN binding protein 2 [Source:HGNC Sym
478	ENSG00000078237	12.157616	30.5997851	-1.3229656	0.04010141	TIGAR	12	protein_coding	TP53 induced glycolysis regulatory phosp
479	ENSG00000174586	47.6141221	96.7371475	-1.0208102	0.04013337	ZNF497	19	protein_coding	zinc finger protein 497 [Source:HGNC Syn
480	ENSG00000212385	1.39E-17	5.92165277	-5.5965168	0.04096458	RNU6-817P	1	snRNA	RNA, U6 small nuclear 817, pseudogene

481	ENSG00000232519	1.39E-17	5.92165277	-5.5965168	0.04096458	AL353807.2	1	antisense	novel transcript, antisense to MSTO1
482	ENSG00000265706	1.39E-17	5.92165277	-5.5965168	0.04096458	SNORD53B	2	snoRNA	small nucleolar RNA, C/D box 53B [Source:HGNC Symbol;Ac
483	ENSG00000272735	1.39E-17	5.92165277	-5.5965168	0.04096458	AC007881.3	2	lincRNA	novel transcript
484	ENSG00000188687	1.39E-17	5.92165277	-5.5965168	0.04096458	SLC4A5	2	protein_coding	solute carrier family 4 member 5 [Source:HGNC Symbol;Acc:
485	ENSG00000235947	1.39E-17	5.92165277	-5.5965168	0.04096458	EGOT	3	lincRNA	eosinophil granule ontogeny transcript [Source:HGNC Symb
486	ENSG00000234737	1.39E-17	5.92165277	-5.5965168	0.04096458	KRT18P15	3	processed_pseud	keratin 18 pseudogene 15 [Source:HGNC Symbol;Acc:HGNC:
487	ENSG00000242992	1.39E-17	5.92165277	-5.5965168	0.04096458	FTH1P4	3	processed_pseud	ferritin heavy chain 1 pseudogene 4 [Source:HGNC Symbol;A
488	ENSG00000145335	1.39E-17	5.92165277	-5.5965168	0.04096458	SNCA	4	protein_coding	synuclein alpha [Source:HGNC Symbol;Acc:HGNC:11138]
489	ENSG00000164136	1.39E-17	5.92165277	-5.5965168	0.04096458	IL15	4	protein_coding	interleukin 15 [Source:HGNC Symbol;Acc:HGNC:5977]
490	ENSG00000271715	1.39E-17	5.92165277	-5.5965168	0.04096458	AC012640.3	5	lincRNA	novel transcript
491	ENSG00000250687	1.39E-17	5.92165277	-5.5965168	0.04096458	AC146944.2	5	transcribed_unpr	NLR family, apoptosis inhibitory protein (NAIP) pseudogene
492	ENSG00000224431	1.39E-17	5.92165277	-5.5965168	0.04096458	AC063976.2	5	processed_pseud	cytoplasmic linker associated protein 2 (CLASP2) pseudoger
493	ENSG00000202533	1.39E-17	5.92165277	-5.5965168	0.04096458	RF00019	5	misc_RNA	
494	ENSG00000285603	1.39E-17	5.92165277	-5.5965168	0.04096458	AL035693.1	6	antisense	novel transcript, antisense to GMDS
495	ENSG00000227758	1.39E-17	5.92165277	-5.5965168	0.04096458	AL645939.2	6	unprocessed_pse	HLA complex group 9 pseudogene 5
496	ENSG00000232757	1.39E-17	5.92165277	-5.5965168	0.04096458	ETF1P1	6	processed_pseud	eukaryotic translation termination factor 1 pseudogene 1 [S
497	ENSG00000223044	1.39E-17	5.92165277	-5.5965168	0.04096458	RNU6-130P	6	snRNA	RNA, U6 small nuclear 130, pseudogene [Source:HGNC Sym
498	ENSG00000227215	1.39E-17	5.92165277	-5.5965168	0.04096458	AL355613.1	6	antisense	novel transcript
499	ENSG00000218233	1.39E-17	5.92165277	-5.5965168	0.04096458	NEPNP	6	unitary_pseudog	nephrocan, pseudogene [Source:HGNC Symbol;Acc:HGNC:3
500	ENSG00000218757	1.39E-17	5.92165277	-5.5965168	0.04096458	AL121952.1	6	processed_pseud	60S ribosomal protein L17 (RPL17) pseudogene
501	ENSG00000226445	1.39E-17	5.92165277	-5.5965168	0.04096458	BX322234.1	6	antisense	uncharacterized LOC101929523 [Source:NCBI gene;Acc:101
502	ENSG00000215045	1.39E-17	5.92165277	-5.5965168	0.04096458	GRID2IP	7	protein_coding	Grid2 interacting protein [Source:HGNC Symbol;Acc:HGNC:1
503	ENSG00000279785	1.39E-17	5.92165277	-5.5965168	0.04096458	AC008267.7	7	TEC	TEC
504	ENSG00000242078	1.39E-17	5.92165277	-5.5965168	0.04096458	AC084864.1	7	lincRNA	novel transcript
505	ENSG00000235262	1.39E-17	5.92165277	-5.5965168	0.04096458	KDM5C-IT1	X	sense_intronic	KDM5C intronic transcript 1 [Source:HGNC Symbol;Acc:HGN
506	ENSG00000228427	1.39E-17	5.92165277	-5.5965168	0.04096458	AL590764.1	X	antisense	uncharacterized LOC107985688 [Source:NCBI gene;Acc:107
507	ENSG00000233260	1.39E-17	5.92165277	-5.5965168	0.04096458	RPSAP63	X	processed_pseud	ribosomal protein SA pseudogene 63 [Source:HGNC Symbol;A
508	ENSG00000246662	1.39E-17	5.92165277	-5.5965168	0.04096458	LINC00535	8	antisense	long intergenic non-protein coding RNA 535 [Source:HGNC S
509	ENSG00000205143	1.39E-17	5.92165277	-5.5965168	0.04096458	ARID3C	9	protein_coding	AT-rich interaction domain 3C [Source:HGNC Symbol;Acc:HG
510	ENSG00000135063	1.39E-17	5.92165277	-5.5965168	0.04096458	FAM189A2	9	protein_coding	family with sequence similarity 189 member A2 [Source:HGN
511	ENSG00000230303	1.39E-17	5.92165277	-5.5965168	0.04096458	AL353743.2	9	lincRNA	novel transcript
512	ENSG00000228069	1.39E-17	5.92165277	-5.5965168	0.04096458	MTCO3P29	9	unprocessed_pse	MT-CO3 pseudogene 29 [Source:HGNC Symbol;Acc:HGNC:52
513	ENSG00000223238	1.39E-17	5.92165277	-5.5965168	0.04096458	RNA5SP294	9	rRNA_pseudogen	RNA, 5S ribosomal pseudogene 294 [Source:HGNC Symbol;A
514	ENSG00000136944	1.39E-17	5.92165277	-5.5965168	0.04096458	LMX1B	9	protein_coding	LIM homeobox transcription factor 1 beta [Source:HGNC Syn
515	ENSG00000255741	1.39E-17	5.92165277	-5.5965168	0.04096458	AP000808.2	11	antisense	novel transcript
516	ENSG00000251323	1.39E-17	5.92165277	-5.5965168	0.04096458	AP003086.1	11	lincRNA	uncharacterized LOC101928865 [Source:NCBI gene;Acc:101
517	ENSG00000256452	1.39E-17	5.92165277	-5.5965168	0.04096458	AP003170.3	11	antisense	novel transcript
518	ENSG00000151963	1.39E-17	5.92165277	-5.5965168	0.04096458	ZNF37CP	10	unprocessed_pse	zinc finger protein 37C, pseudogene [Source:HGNC Symbol;A
519	ENSG00000230609	1.39E-17	5.92165277	-5.5965168	0.04096458	AL513185.2	10	processed_pseud	cytochrome c oxidase subunit VIIc (COX7C) pseudogene
520	ENSG00000278459	1.39E-17	5.92165277	-5.5965168	0.04096458	RF00017	12	misc_RNA	

521	ENSG00000229456	1.39E-17	5.92165277	-5.5965168	0.04096458	RLIMP1	13	processed_pseuc	ring finger protein, LIM domain interacting pseudogene 1 [S
522	ENSG00000229323	1.39E-17	5.92165277	-5.5965168	0.04096458	DLEU1-AS1	13	lincRNA	DLEU1 antisense RNA 1 [Source:HGNC Symbol;Acc:HGNC:50
523	ENSG00000279594	1.39E-17	5.92165277	-5.5965168	0.04096458	AL049780.3	14	TEC	TEC
524	ENSG00000258454	1.39E-17	5.92165277	-5.5965168	0.04096458	AC016526.2	14	sense_overlappir	novel transcript, sense overlapping GPATCH2L
525	ENSG00000258826	1.39E-17	5.92165277	-5.5965168	0.04096458	AL157955.2	14	lincRNA	uncharacterized LOC101928791 [Source:NCBI gene;Acc:101
526	ENSG00000258858	1.39E-17	5.92165277	-5.5965168	0.04096458	AL583722.4	14	antisense	novel transcript, antisense to INF2
527	ENSG00000104044	1.39E-17	5.92165277	-5.5965168	0.04096458	OCA2	15	protein_coding	OCA2 melanosomal transmembrane protein [Source:HGNC S
528	ENSG00000270016	1.39E-17	5.92165277	-5.5965168	0.04096458	AC026150.3	15	antisense	novel transcript, antisense to AC026150.11
529	ENSG00000259845	1.39E-17	5.92165277	-5.5965168	0.04096458	HERC2P10	15	unprocessed_pse	hect domain and RLD 2 pseudogene 10 [Source:HGNC Symb
530	ENSG00000274444	1.39E-17	5.92165277	-5.5965168	0.04096458	AC078909.2	15	lincRNA	novel transcript
531	ENSG00000275672	1.39E-17	5.92165277	-5.5965168	0.04096458	AC025580.3	15	antisense	GATM antisense RNA 1
532	ENSG00000157470	1.39E-17	5.92165277	-5.5965168	0.04096458	FAM81A	15	protein_coding	family with sequence similarity 81 member A [Source:HGNC
533	ENSG00000259649	1.39E-17	5.92165277	-5.5965168	0.04096458	AC027808.2	15	antisense	uncharacterized LOC107984749 [Source:NCBI gene;Acc:107
534	ENSG00000259257	1.39E-17	5.92165277	-5.5965168	0.04096458	AC037479.1	15	processed_pseuc	Coiled-coil domain-containing protein 117 (CC117) pseudog
535	ENSG00000279136	1.39E-17	5.92165277	-5.5965168	0.04096458	AL031258.1	16	TEC	TEC
536	ENSG00000261613	1.39E-17	5.92165277	-5.5965168	0.04096458	AC093525.6	16	antisense	novel transcript, antisense to PDPK1
537	ENSG00000261583	1.39E-17	5.92165277	-5.5965168	0.04096458	AC012317.1	16	antisense	novel transcript
538	ENSG00000260448	1.39E-17	5.92165277	-5.5965168	0.04096458	LCMT1-AS1	16	bidirectional_pro	LCMT1 antisense RNA 1 [Source:HGNC Symbol;Acc:HGNC:51
539	ENSG00000270165	1.39E-17	5.92165277	-5.5965168	0.04096458	AC010530.1	16	sense_intronic	novel transcript
540	ENSG00000260156	1.39E-17	5.92165277	-5.5965168	0.04096458	AC020763.1	16	antisense	novel transcript, antisense to VAC14
541	ENSG00000263219	1.39E-17	5.92165277	-5.5965168	0.04096458	RYKP1	17	processed_pseuc	receptor-like tyrosine kinase pseudogene 1 [Source:HGNC S
542	ENSG00000261868	1.39E-17	5.92165277	-5.5965168	0.04096458	MFSD1P1	17	processed_pseuc	major facilitator superfamily domain containing 1 pseudoge
543	ENSG00000225751	1.39E-17	5.92165277	-5.5965168	0.04096458	AC087501.1	17	antisense	novel transcript, antisense to STX8
544	ENSG00000108452	1.39E-17	5.92165277	-5.5965168	0.04096458	ZNF29P	17	unprocessed_pse	zinc finger protein 29, pseudogene [Source:HGNC Symbol;Ac
545	ENSG00000221540	1.39E-17	5.92165277	-5.5965168	0.04096458	MIR1180	17	miRNA	microRNA 1180 [Source:HGNC Symbol;Acc:HGNC:35261]
546	ENSG00000266371	1.39E-17	5.92165277	-5.5965168	0.04096458	AC079915.1	17	sense_intronic	novel transcript, sense intronic NF1
547	ENSG00000262759	1.39E-17	5.92165277	-5.5965168	0.04096458	MRPS21P9	17	processed_pseuc	mitochondrial ribosomal protein S21 pseudogene 9 [Source:I
548	ENSG00000280233	1.39E-17	5.92165277	-5.5965168	0.04096458	AC015813.7	17	TEC	novel transcript
549	ENSG00000267302	1.39E-17	5.92165277	-5.5965168	0.04096458	RNFT1-DT	17	lincRNA	RNFT1 divergent transcript [Source:HGNC Symbol;Acc:HGNC
550	ENSG00000266943	1.39E-17	5.92165277	-5.5965168	0.04096458	RPSAP66	17	processed_pseuc	ribosomal protein SA pseudogene 66 [Source:HGNC Symbol;
551	ENSG00000215644	1.39E-17	5.92165277	-5.5965168	0.04096458	GCGR	17	protein_coding	glucagon receptor [Source:HGNC Symbol;Acc:HGNC:4192]
552	ENSG00000199719	1.39E-17	5.92165277	-5.5965168	0.04096458	RN7SKP74	20	misc_RNA	RNA, 7SK small nuclear pseudogene 74 [Source:HGNC Symb
553	ENSG00000278367	1.39E-17	5.92165277	-5.5965168	0.04096458	AL356652.1	20	antisense	novel transcript, antisense to PROCR
554	ENSG00000252933	1.39E-17	5.92165277	-5.5965168	0.04096458	RNU6-1223P	19	snRNA	RNA, U6 small nuclear 1223, pseudogene [Source:HGNC Syn
555	ENSG00000267611	1.39E-17	5.92165277	-5.5965168	0.04096458	AC008521.1	19	processed_pseuc	MAD2 mitotic arrest deficient-like 1 (yeast) (MAD2L1) pseu
556	ENSG00000232677	1.39E-17	5.92165277	-5.5965168	0.04096458	LINC00665	19	lincRNA	long intergenic non-protein coding RNA 665 [Source:HGNC S
557	ENSG00000171777	1.39E-17	5.92165277	-5.5965168	0.04096458	RASGRP4	19	protein_coding	RAS guanyl releasing protein 4 [Source:HGNC Symbol;Acc:H
558	ENSG00000267375	1.39E-17	5.92165277	-5.5965168	0.04096458	AC008649.2	19	antisense	novel transcript, antisense to ACTN4
559	ENSG00000283673	1.39E-17	5.92165277	-5.5965168	0.04096458	MIR4531	19	miRNA	microRNA 4531 [Source:HGNC Symbol;Acc:HGNC:41872]
560	ENSG00000105467	1.39E-17	5.92165277	-5.5965168	0.04096458	SYNGR4	19	protein_coding	synaptogyrin 4 [Source:HGNC Symbol;Acc:HGNC:11502]

561	ENSG00000221241	1.39E-17	5.92165277	-5.5965168	0.04096458	SNORD88A	19	snoRNA	small nucleolar RNA, C/D box 88A [Source:HGNC Symbol;Acc
562	ENSG00000207780	1.39E-17	5.92165277	-5.5965168	0.04096458	MIR648	22	miRNA	microRNA 648 [Source:HGNC Symbol;Acc:HGNC:32904]
563	ENSG00000281530	1.39E-17	5.92165277	-5.5965168	0.04096458	AC004461.2	22	sense_intronic	DiGeorge syndrome critical region gene 12 (non-protein cod
564	ENSG00000229955	1.39E-17	5.92165277	-5.5965168	0.04096458	Z98749.1	22	sense_intronic	novel transcript, sense intronic to CSNK1E
565	ENSG00000237609	1.39E-17	5.92165277	-5.5965168	0.04096458	AF064858.2	21	lincRNA	novel transcript
566	ENSG00000271732	2.02720959	10.8572834	-2.3521894	0.04103203	AL137798.1	1	lincRNA	novel transcript
567	ENSG00000227692	2.02720959	10.8572834	-2.3521894	0.04103203	MED28P3	2	processed_pseud	mediator complex subunit 28 pseudogene 3 [Source:HGNC S
568	ENSG00000238228	2.02720959	10.8572834	-2.3521894	0.04103203	OR7E7P	7	unprocessed_pse	olfactory receptor family 7 subfamily E member 7 pseudoger
569	ENSG00000254208	2.02720959	10.8572834	-2.3521894	0.04103203	AC011773.1	8	antisense	novel transcript, antisense to E2F5
570	ENSG00000232684	2.02720959	10.8572834	-2.3521894	0.04103203	ATP11A-AS1	13	antisense	ATP11A antisense RNA 1 [Source:HGNC Symbol;Acc:HGNC:4
571	ENSG00000258458	2.02720959	10.8572834	-2.3521894	0.04103203	AL160314.2	14	processed_transc	novel transcript, antisense to OXA1L
572	ENSG00000264049	2.02720959	10.8572834	-2.3521894	0.04103203	MIR4737	17	miRNA	microRNA 4737 [Source:HGNC Symbol;Acc:HGNC:41669]
573	ENSG00000143375	18.2358728	42.4452835	-1.2133353	0.04153513	CGN	1	protein_coding	cingulin [Source:HGNC Symbol;Acc:HGNC:17429]
574	ENSG00000125966	18.2358728	42.4452835	-1.2133353	0.04153513	MMP24	20	protein_coding	matrix metallopeptidase 24 [Source:HGNC Symbol;Acc:HGNC
575	ENSG00000260750	10.1315311	26.6512854	-1.3846269	0.04190398	AC092720.1	16	lincRNA	uncharacterized LOC101928737 [Source:NCBI gene;Acc:101ç
576	ENSG00000253958	9.11848879	24.6770356	-1.424185	0.04281416	CLDN23	8	protein_coding	claudin 23 [Source:HGNC Symbol;Acc:HGNC:17591]
577	ENSG00000164764	9.11848879	24.6770356	-1.424185	0.04281416	SBSPON	8	protein_coding	somatomedin B and thrombospondin type 1 domain containi
578	ENSG00000258604	9.11848879	24.6770356	-1.424185	0.04281416	AL161668.4	14	lincRNA	novel transcript
579	ENSG00000166869	9.11848879	24.6770356	-1.424185	0.04281416	CHP2	16	protein_coding	calcineurin like EF-hand protein 2 [Source:HGNC Symbol;Acc
580	ENSG00000146094	37.4836908	77.9817766	-1.0544438	0.04340952	DOK3	5	protein_coding	docking protein 3 [Source:HGNC Symbol;Acc:HGNC:24583]
581	ENSG00000214967	22.2880447	49.3551573	-1.1426138	0.04363981	NPIPA7	16	protein_coding	nuclear pore complex interacting protein family member A7
582	ENSG00000229539	16.209787	37.5096592	-1.2042531	0.04368423	AL353194.1	20	antisense	novel transcript
583	ENSG00000235750	8.10544673	22.7027856	-1.472011	0.04369299	KIAA0040	1	protein_coding	KIAA0040 [Source:HGNC Symbol;Acc:HGNC:28950]
584	ENSG00000204380	8.10544673	22.7027856	-1.472011	0.04369299	PKP4-AS1	2	antisense	PKP4 antisense RNA 1 [Source:HGNC Symbol;Acc:HGNC:525ε
585	ENSG00000205090	3.04024516	12.8315343	-2.033924	0.04399131	TMEM240	1	protein_coding	transmembrane protein 240 [Source:HGNC Symbol;Acc:HGN(
586	ENSG00000270392	3.04024516	12.8315343	-2.033924	0.04399131	PFN1P2	1	transcribed_proc	profilin 1 pseudogene 2 [Source:HGNC Symbol;Acc:HGNC:24
587	ENSG00000187862	3.04024516	12.8315343	-2.033924	0.04399131	TTC24	1	protein_coding	tetratricopeptide repeat domain 24 [Source:HGNC Symbol;Ac
588	ENSG00000276334	3.04024516	12.8315343	-2.033924	0.04399131	AL133243.2	2	sense_intronic	novel transcript, sense intronic to BIRC6
589	ENSG00000235852	3.04024516	12.8315343	-2.033924	0.04399131	AC005540.1	2	antisense	novel transcript
590	ENSG00000102057	3.04024516	12.8315343	-2.033924	0.04399131	KCND1	X	protein_coding	potassium voltage-gated channel subfamily D member 1 [So
591	ENSG00000107099	3.04024516	12.8315343	-2.033924	0.04399131	DOCK8	9	protein_coding	dedicator of cytokinesis 8 [Source:HGNC Symbol;Acc:HGNC:1
592	ENSG00000129749	3.04024516	12.8315343	-2.033924	0.04399131	CHRNA10	11	protein_coding	cholinergic receptor nicotinic alpha 10 subunit [Source:HGN(
593	ENSG00000200090	3.04024516	12.8315343	-2.033924	0.04399131	RF00019	11	misc_RNA	
594	ENSG00000215146	3.04024516	12.8315343	-2.033924	0.04399131	BX322639.1	10	transcribed_unpr	zinc finger protein 91 pseudogene [Source:NCBI gene;Acc:44
595	ENSG00000256185	3.04024516	12.8315343	-2.033924	0.04399131	AC055720.2	12	3prime_overlappi	novel transcript
596	ENSG00000272848	7.09240497	20.7285356	-1.5310368	0.04448909	AL135910.1	6	lincRNA	novel transcript
597	ENSG00000261067	7.09240497	20.7285356	-1.5310368	0.04448909	AC109460.3	16	processed_transc	novel transcript
598	ENSG00000196569	6.07936364	18.7542855	-1.6057905	0.04511747	LAMA2	6	protein_coding	laminin subunit alpha 2 [Source:HGNC Symbol;Acc:HGNC:64ε
599	ENSG00000278390	6.07936364	18.7542855	-1.6057905	0.04511747	AL354696.2	13	antisense	uncharacterized LOC101929140 [Source:NCBI gene;Acc:101ç
600	ENSG00000260367	6.07936364	18.7542855	-1.6057905	0.04511747	AC109460.1	16	antisense	novel transcript, antisense to SPNS1

601	ENSG00000183054	4.05328324	14.8057849	-1.8377989	0.04520218	RGPD6	2	protein_coding	RANBP2-like and GRIP domain containing 6 [Source:HGNC Sy
602	ENSG00000248103	4.05328324	14.8057849	-1.8377989	0.04520218	AC008443.2	5	lincRNA	novel transcript
603	ENSG00000261553	4.05328324	14.8057849	-1.8377989	0.04520218	AL137782.1	13	processed_transc	novel transcript
604	ENSG00000006047	4.05328324	14.8057849	-1.8377989	0.04520218	YBX2	17	protein_coding	Y-box binding protein 2 [Source:HGNC Symbol;Acc:HGNC:179
605	ENSG00000268543	4.05328324	14.8057849	-1.8377989	0.04520218	AC012313.4	19	TEC	novel transcript
606	ENSG00000224959	5.06632294	16.7800353	-1.7036813	0.04543596	AC017002.1	2	lincRNA	novel transcript
607	ENSG00000261051	5.06632294	16.7800353	-1.7036813	0.04543596	AC107021.2	3	sense_overlappir	novel transcript, overlapping to PLOD2
608	ENSG00000184923	5.06632294	16.7800353	-1.7036813	0.04543596	NUTM2A	10	protein_coding	NUT family member 2A [Source:HGNC Symbol;Acc:HGNC:234
609	ENSG00000197813	5.06632294	16.7800353	-1.7036813	0.04543596	AC011450.1	19	lincRNA	novel transcript
610	ENSG00000239665	35.4576046	72.0590278	-1.0205737	0.0460164	AL157392.3	10	processed_transc	novel transcript
611	ENSG00000203709	38.4967339	78.9689014	-1.0342143	0.04666866	MIR29B2CHG	1	lincRNA	MIR29B2 and MIR29C host gene [Source:HGNC Symbol;Acc:H
612	ENSG00000204524	42.5489064	85.8787749	-1.0111011	0.04718722	ZNF805	19	protein_coding	zinc finger protein 805 [Source:HGNC Symbol;Acc:HGNC:2327
613	ENSG00000225422	24.3141307	52.3165317	-1.1016116	0.04740969	RBMS1P1	12	processed_pseud	RNA binding motif single stranded interacting protein 1 pseu
614	ENSG00000170092	13.1706587	31.58691	-1.2542385	0.04754413	SPDYE5	7	protein_coding	speedy/RINGO cell cycle regulator family member E5 [Source
615	ENSG00000157999	18.2358728	40.4710338	-1.1448291	0.04859715	ANKRD61	7	protein_coding	ankyrin repeat domain 61 [Source:HGNC Symbol;Acc:HGNC:2
616	ENSG00000233230	32.4184753	66.136279	-1.02587	0.04913111	AC079807.1	2	antisense	novel transcript
617	ENSG00000214855	32.4184753	66.136279	-1.02587	0.04913111	APOC1P1	19	transcribed_unpr	apolipoprotein C1 pseudogene 1 [Source:HGNC Symbol;Acc:H
